# Photo- and thermo-responsive complementary metal coordination for the construction of supramolecular polymers

**DOI:** 10.1039/d6sc02381a

**Published:** 2026-05-07

**Authors:** Shenghui Rao, Yuzhen Wang, Ju-An Zhang, Liqing Li, Shaoyu Li, Linlin Liu, Yu Xie, Hui Li, Wei Tian

**Affiliations:** a Jiangxi Province Key Laboratory of Functional Crystalline Materials Chemistry, School of Chemistry and Chemical Engineering, Jiangxi University of Science and Technology Ganzhou 341000 Jiangxi Province P. R. China lh@jxust.edu.cn; b Shaanxi Key Laboratory of Macromolecular Science and Technology, School of Chemistry and Chemical Engineering, Northwestern Polytechnical University Xi'an 710072 P. R. China happytw_3000@nwpu.edu.cn; c College of Environment and Chemical Engineering, Nanchang Hangkong University Nanchang 330063 P. R. China xieyu_121@163.com

## Abstract

Among diverse metal coordination interactions, complementary metal–ligand coordination stands out as a significant category, featuring captivating coordination geometries and excellent binding capacity. Although some complementary ligand pairs have been developed, few studies have focused on the regulation of their coordination structures or the exploration of synergistic effects between different coordination motifs. Herein, we report the reversible structural regulation of terpyridyl-based complementary metal coordination *via* photooxidation and thermal reduction, and construct fluorescent supramolecular polymers (FSLP) by integrating dual synergistic complementary metal coordination interactions with host–guest recognition. Photooxidation of the complementary metal coordination enables its disassembly, while heating induces its reformation, thereby realizing reversible degradation and regeneration of the FSLP. Given the fascinating coordination structures and photo- and thermo-responsive properties, this study provides inspiration for designing new functional supramolecular assemblies.

## Abstract 2

This study investigated the photo- and thermo-responsive modulation of terpyridine-based complementary metal coordination, as well as synergistic effects between different complementary coordination motifs.

## Graphical abstract

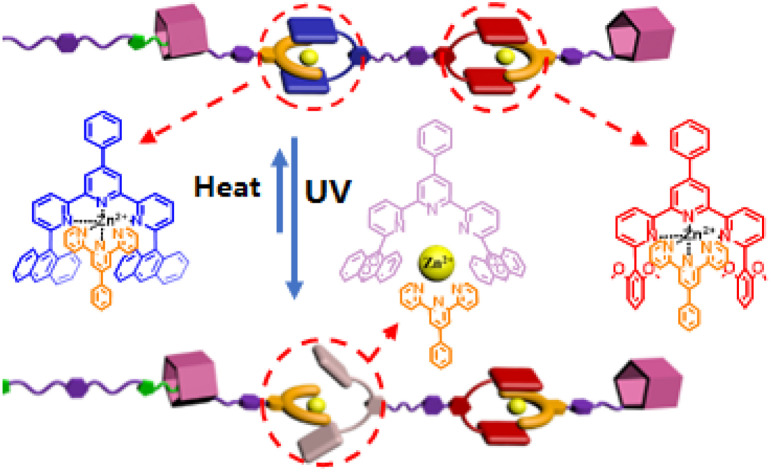

## Introduction

The formation of biomacromolecules relies on the role of self-assembly. Natural biomacromolecules such as amino acids, peptides, proteins, and DNA, constructed through collaborative self-assembly, perform important biological functions.^1^ Drawing inspiration from this bottom-up assembly of biomacromolecules, chemists are devoted to developing new strategies to construct complex supramolecular assemblies that mimic the structures and functions of biomacromolecules.^2–10^ The construction of supramolecular assemblies mainly depends on various noncovalent interactions. Among these noncovalent interactions, metal coordination has gained favor due to its abundance, directionality, and tunable coordination strength, making it an ideal candidate for applications in a wide range of fields.^11–21^ Currently, supramolecular assemblies driven by metal coordination are gradually progressing toward increased structural complexity and functionalization.^22,23^ Challenges remain in exercising precise control over supramolecular assembly structures and gaining a deeper understanding of the structure–function relationship.

**Scheme 1:**
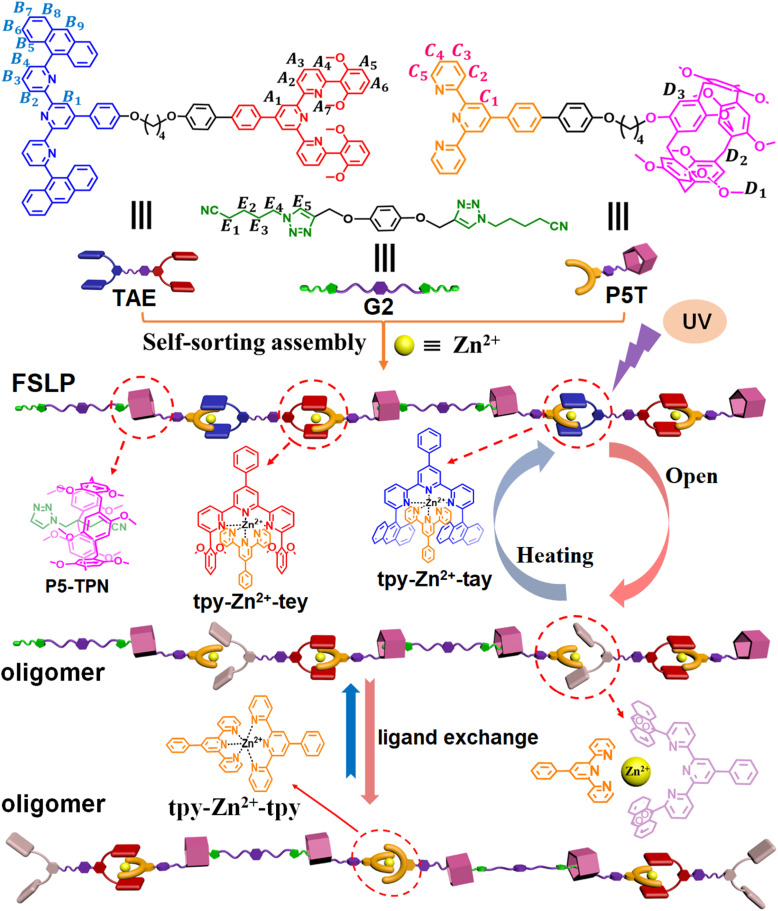
Schematic representation of the FSLP constructed from TAE + P5T + G2 + Zn(OTf)_2_*via* self-sorting assembly, as well as its regulable photochemical reaction triggered by UV irradiation or heating.

Terpyridine (tpy) and its derivatives, as an important class of ligands, can coordinate with various metal ions to form complexes with different coordination strengths.^24–29^ Nevertheless, when such ligands are employed for fabricating complex multicomponent supramolecular assemblies, unforeseen errors often occur during assembly.^30^ Achieving precise supramolecular assembly in multi-component systems remains challenging. To address these issues, some complementary ligand pairs have been developed that can form high-fidelity, thermodynamically or kinetically stable coordination structures with metal ions.^31–33^ These complementary coordination reactions are governed by two synergistic mechanisms: (i) the intrinsic geometric compatibility between ligands and metal ion centers, and (ii) π–π stacking interactions are established between the ligand substituents and the complementary ligands. These cooperative effects enable the quantitative formation of heteroleptic complexes with precise stoichiometric control, significantly enhancing the predictability of supramolecular assembly. Although these complementary ligand pairs exhibit remarkable assembly selectivity and advantages, the investigation of synergistic effects between different complementary metal coordination interactions for constructing supramolecular assemblies remains unexplored to date. Moreover, the modulation of their coordination structures, especially *via* photochemical interactions that reversibly control their coordination behavior, has not yet been studied. Herein, we report the photo/thermo-responsive regulation of tpy-based complementary metal coordination and demonstrate the construction of supramolecular polymers by integrating dual synergistic complementary coordination interactions with host–guest recognition (Scheme 1). The coordination structure and resulting supramolecular polymer exhibit several characteristics: (1) the complementary metal coordination forms thermodynamically stable “sandwich” structures, unlike the labile homoleptic coordination tpy-M-tpy, which is affected by uncontrollable ligand scrambling. (2) The complementary coordination structure can undergo reversible disassembly and reformation through a controllable photoreaction or heating. (3) The supramolecular polymer fabricated *via* dual synergistic complementary coordination interactions with host–guest recognition show regulatable fluorescence emission by varying pH, solvent polarity, light irradiation, and heating.

## Results and discussion

**Scheme 2:**
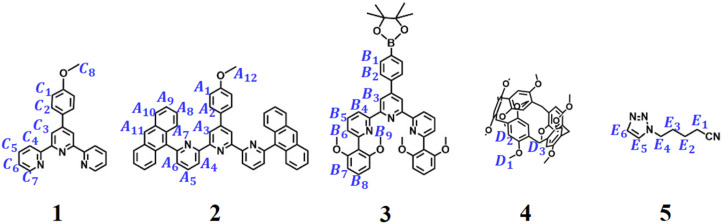
Molecular structures of the model compounds 1–5.

Three distinct monomers TAE, P5T, and G2 were designed and synthesized (Scheme 1). Monomer TAE incorporates a 6,6″-di(2,6-dimethoxyphenyl)-substituted terpyridine moiety (tey) and a 6,6″-anthracyl-substituted terpyridine moiety (tay). Monomer P5T comprises a terpyridine unit (tpy) covalently linked to a pillar[5]arene group (P5), while monomer G2 features two neutral guest groups (TPN). To elucidate the self-sorting assembly mechanism of these monomers, five model compounds were synthesized (1–5, Scheme 2). Different mixtures of model compounds were prepared in CDCl_3_–CD_3_COCD_3_ (3 : 1, v/v) for ^1^H NMR analysis. The ^1^H NMR of 1 + Zn(OTf)_2_ confirmed the homoleptic tpy-Zn^2+^-tpy coordination through the chemical shift analysis of terpyridine protons (Fig. S1a). In contrast, 2 + Zn(OTf)_2_ only exhibited partial coordination, as indicated by peak integration analysis (Fig. S1e), which is attributable to the steric hindrance from the anthracene substituents.^30^ The ^1^H NMR of 3 + Zn(OTf)_2_ also showed only partial coordination between 3 and zinc ion due to the steric hindrance caused by the bulky dimethoxyphenyl groups (Fig. S3e). The ^1^H NMR and ^1^H–^1^H COSY NMR of 1 + 2 + Zn(OTf)_2_ exhibited noticeable variations in the chemical shifts of protons compared to those of 1 + Zn(OTf)_2_ and 2 + Zn(OTf)_2_, suggesting significant alterations in their coordination species (Fig. S1c and S2). Protons C3/C5 on compound 1 and A7–A8 on compound 2 exhibited pronounced upfield shifts, while A3–A4 protons on compound 2 showed downfield shifts. These observations, coupled with the high-resolution ESI-MS data of 1 + 2 + Zn(OTf)_2_ (*m*/*z* = 547.1633, Fig. S3a), confirmed the formation of a heteroleptic tpy-Zn^2+^-tay coordination complex. Similar to the ^1^H NMR of 1 + 2 + Zn(OTf)_2_, the ^1^H NMR and ^1^H–^1^H COSY NMR of 1 + 3 + Zn(OTf)_2_ clearly showed significant upfield shifts for protons C3 on 1 and B6–B8 on 3, while downfield shifts for protons B3–B4 on 3 were observed (Fig. S4c and S5). These changes in chemical shifts can be attributed to the π–π stacking interactions between the pyridine group and the 2,6-dimethoxyphenyl substituents, signifying that 1 + 3 + Zn(OTf)_2_ is capable of forming a heteroleptic tpy-Zn^2+^-tey metal coordination with a sandwich-like structure.^31^ The high-resolution ESI-mass spectrum of 1 + 3 + Zn(OTf)_2_ further provided direct evidence for the formation of tpy-Zn^2+^-tey at *m*/*z* = 555.1931 (Fig. S3b). In contrast, the ^1^H NMR of 2 + 3 + Zn(OTf)_2_ reveals that the mixture includes heteroleptic coordination complex, homoleptic coordination complex, and uncoordinated ligands due to the steric hindrance caused by the large anthracyl and dimethoxyphenyl groups on 2 and 3 (Fig. S6d). Furthermore, when 1 + 2 + 3 + Zn(OTf)_2_ were mixed together, the ^1^H NMR clearly demonstrated the self-sorting complexation between tpy-Zn^2+^-tey and tpy-Zn^2+^-tay (Fig. S6c). On the other hand, the successful formation of host–guest complex P5–TPN was evidenced by analyzing the ^1^H NMR of 4 + 5 (Fig. S7), indicating the effective incorporation of neutral guest TPN into the cavity of host P5.^34–36^ Ultimately, the ^1^H NMR analysis of 1 + 2 + 3 + 4 + 5 + Zn(OTf)_2_ verified that self-sorting complexation occurred among tpy-Zn^2+^-tay, tpy-Zn^2+^-tey, and P5–TPN in CDCl_3_–CD_3_COCD_3_ (3 : 1, v/v, Fig. S8).

**Fig. 1:**
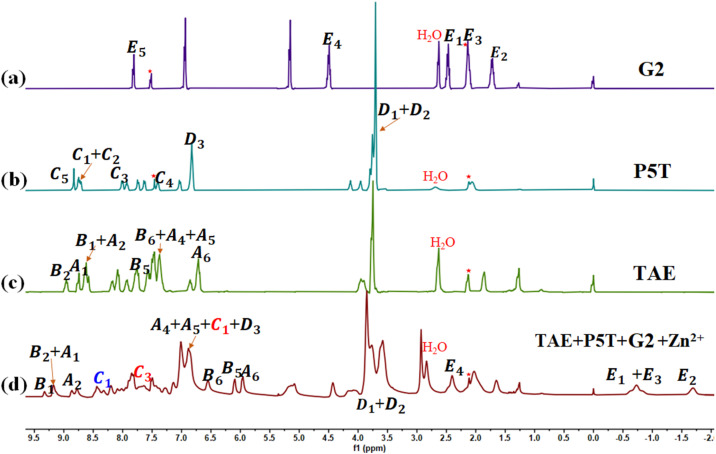
The ^1^H NMR of (a) G2, (b) P5T, (c) TAE, and (d) TAE + P5T + G2 + Zn(OTf)_2_ in CDCl_3_–CD_3_COCD_3_ (v/v = 3 : 1, molar ratio: TAE : P5T : G2 : Zn(OTf)_2_ = 1 : 2 : 1 : 2).

The self-assembly of TAE + P5T + G2 + Zn(OTf)_2_ was subsequently investigated by mixing TAE, P5T, G2, and Zn(OTf)_2_ in a 1 : 2 : 1 : 2 molar ratio and dissolving the mixture in CDCl_3_–CD_3_COCD_3_ (3 : 1, v/v). The presence of multiple noncovalent interactions led to increased complexity in the ^1^H NMR of TAE + P5T + G2 + Zn(OTf)_2_ (Fig. 1d). The chemical shifts of protons were assigned using ^1^H–^1^H COSY NMR (Fig. S9) and by reference to the ^1^H NMR spectra of model compounds. The ^1^H NMR spectra of model compounds. Notably, the ^1^H NMR spectrum showed significant upfield shifts for protons E1–E4 (2.40, −0.74, −0.79, −1.69 ppm). Furthermore, a strong correlation was observed between protons E1–E4 on G2 and D1–D3 on P5T in the NOESY spectrum (Fig. S10), indicating full incorporation of the TPN moiety into the cavity of the P5 group on P5T, which results in tight P5–TPN binding.^36^ Proton C1 in the tpy group of P5T exhibits two distinct chemical shifts in the ^1^H NMR of TAE + P5T + G2 + Zn(OTf)_2_ (Fig. 1d), which can be attributed to the presence of two different complementary metal coordination interactions tpy-Zn^2+^-tay and tpy-Zn^2+^-tey, as indicated by different colors (red and blue) in the ^1^H NMR spectrum. Protons B1–B2 on TAE underwent a downfield shift, while proton C1 (highlighted in blue) on P5T and protons B5–B6 on TAE exhibited upfield shifts (Fig. 1d). 2D NOESY analysis of TAE + P5T + G2 + Zn(OTf)_2_ revealed the correlation between protons B5–B6 on TAE and proton C2 on P5T (Fig. S10). Both the ^1^H NMR and 2D NOESY spectra confirmed the presence of a heteroleptic metal coordination (specifically tpy-Zn^2+^-tay) between TAE and P5T. Similarly, protons A1 on TAE underwent a downfield shift (Fig. 1d), while proton C1 (highlighted in red), proton C3 on P5T, and protons A5–A6 on TAE shifted upfield. A correlation between protons A6–A7 on TAE and proton C2 on P5T was also observed in the 2D NOESY spectrum (Fig. S10). Both the ^1^H NMR and 2D NOESY spectra confirmed the formation of another heteroleptic metal coordination structure (tpy-Zn^2+^-tey) between TAE and P5T. Thus, the mixture of TAE, P5T, G2, and Zn(OTf)_2_ underwent self-sorting assembly to form an alternating supramolecular array, represented as [P5T–TAE–P5T–G2]_*n*_. Concentration-dependent ^1^H NMR spectroscopy verified that the ^1^H NMR signals of TAE + P5T + G2 + Zn(OTf)_2_ gradually broadened with increasing monomer concentration (Fig. S11), indicating the formation of fluorescent supramolecular polymers (FSLP) at high concentration.^37,38^

**Fig. 2:**
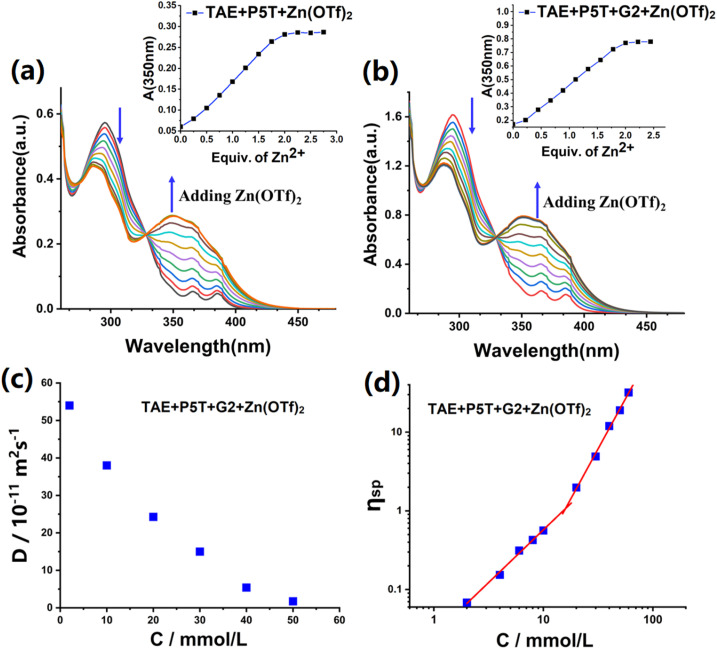
UV-vis absorbance variations upon sequential addition of zinc ions to (a) TAE + P5T (0.02 mM, TAE : P5T = 1 : 2), (b) TAE + P5T + G2 (0.02 mM, TAE : P5T : G2 = 1 : 2 : 1). Inset of (a and b) showed normalized absorbance changes at 350 nm, respectively, (c) average diffusion coefficient of TAE + P5T + G2 + Zn(OTf)_2_ against the concentration of TAE (CDCl_3_–CD_3_COCD_3_ = 3/1, v/v), (d) effect of concentration on the specific viscosity of TAE + P5T + G2 + Zn(OTf)_2_. Molar ratio: TAE : P5T : G2 : Zn(OTf)_2_ = 1 : 2 : 1 : 2.

The self-sorting assembly process of TAE + P5T + G2 + Zn(OTf)_2_ was analyzed using the UV-vis titration technique. Experimental samples containing model compounds 1, 1 + 2, and 1 + 3 were prepared and subsequently titrated with Zn(OTf)_2_. As depicted in Fig. S12a–c, all three systems (1, 1 + 2, and 1 + 3) exhibited characteristic absorption profiles in their titration curves, with a prominent band near 285 nm arising from π–π* electronic transition. As the concentration of zinc ions increased, a new absorption peak developed at approximately 350 nm in the titration curves, while the absorption bands near 285 nm gradually diminished. The experimental data indicated a progressive conversion of free ligands into metal-coordinated species through sequential titration.^39^ Furthermore, the absorbance near 350 nm reached its maximum at a Zn^2+^-to-ligand molar ratio of 1 : 2. These changes verified the formation of the tpy-Zn^2+^-tpy coordination complex for the solution of 1 + Zn(OTf)_2_, the tpy-Zn^2+^-tay coordination complex for the solution of 1 + 2 + Zn(OTf)_2_, and the tpy-Zn^2+^-tey coordination complex for the solution of 1 + 3 + Zn(OTf)_2_, respectively. Furthermore, in the titration of 1 + 2, we also observed that the three characteristic absorption bands of anthracene in the range of 340–390 nm gradually disappeared (Fig. S12a), further implying that the complementary coordination structure of tpy-Zn^2+^-tay had been formed. When Zn(OTf)_2_ was titrated into the mixed solution of 1 + 2 + 3 (with a molar ratio of 1 : 2 : 3 = 2 : 1 : 1), the resulting titration curve exhibited similarities to the combined titration curves of 1 + 2 and 1 + 3 (Fig. S12d). The maximum absorbance band was obtained when the molar ratio of components 1, 2, 3, and Zn(OTf)_2_ was adjusted to 2 : 1 : 1 : 2. These findings unequivocally indicate that the two distinct metal coordination interactions, namely tpy-Zn^2+^-tay and tpy-Zn^2+^-tey, coexist in the solution of 1 + 2 + 3 + Zn(OTf)_2_. In contrast, adding zinc ions to the equimolar mixture of 2 + 3 resulted in only a slight diminution in the absorption intensity at 292 nm on the titration profile of 2 + 3 (Fig. S12e). This observation further confirms the difficulty in forming tay-Zn^2+^-tay, tey-Zn^2+^-tey, and tay-Zn^2+^-tey complexes due to significant steric hindrance from both tay and tey groups, consistent with the results of the aforementioned ^1^H NMR of 2 + 3 + Zn(OTf)_2_. The titration curve of TAE + P5T (with a molar ratio of TAE : P5T = 1 : 2), as depicted in Fig. 2a, closely matched that of the model compounds 1 + 2 + 3. During zinc ion titration, the absorption intensity at 290 nm decreased progressively, and a new absorption band developed at 350 nm. When the molar ratio of TAE, P5T, and Zn(OTf)_2_ was adjusted to 1 : 2 : 2, the absorbance band located at 350 nm reached its peak value. The titration curve of TAE + P5T + G2 (with a molar ratio of TAE : P5T : G2 = 1 : 2 : 1) was similar to that of TAE + P5T (Fig. 2b), demonstrating the self-sorting complexation among the tpy-Zn^2+^-tay, tpy-Zn^2+^-tey, and P5–TPN noncovalent interactions during self-assembly.

2D DOSY NMR was implemented to further investigate the supramolecular self-assembly. In the solution of TAE + P5T + G2 + Zn(OTf)_2_ (molar ratio TAE : P5T : G2 = 1 : 2 : 1), DOSY measurements revealed concentration-dependent characteristic, with diffusion coefficients decreasing from 5.42 × 10^−10^ m^2^ s^−1^ (2 mM) to 1.73 × 10^−11^ m^2^ s^−1^ (50 mM). This 31-fold reduction (*D*_2.0mM_/*D*_50.0mM_ = 31) further corroborates the structural evolution from discrete oligomeric species to extended supramolecular polymers with increasing monomer concentrations (Fig. 2c and S13).^40,41^ Viscosities were measured to provide additional insight into the supramolecular polymerization. The viscosity–concentration profile exhibits a slope of 1.33 at low monomer concentrations, which increases to 2.75 with increasing monomer concentration (Fig. 2d). This nonlinear increasement in slope provides evidence for the transition from discrete supramolecular oligomers into larger polymeric assemblies.^42^ TEM was used to observe the morphological features of supramolecular assemblies.

The representative TEM micrograph shows spherical nanostructures of FSLP (Fig. S14). It is worth noting that, due to microscopic technical constraints, it is difficult for TEM to observe the intrinsic chain structures of supramolecular polymers at the molecular level. When FSLP is transferred from solution to a dry state, the dynamic characteristics of supramolecular polymerization may trigger the secondary assembly of FSLP. Since both TAE and P5T monomers contain flexible alkyl chains, the globular morphology is presumably formed by interchain entanglement and molecular folding. Similar spherical morphologies have also been observed in existing studies on linear supramolecular polymers.^34^

Next, the fluorescence characteristics of FSLP were investigated. Given that monomer P5T features a more extended π-conjugated system of benzene rings than model compound 1, the P5T + 3, P5T + 2, and P5T + 2 + 3 complexes were selected as fluorescence titration targets. Zn(OTf)_2_ was incrementally added to the respective solutions to generate titration profiles. Upon the addition of Zn^2+^, a significant redshift of emission band accompanied by intensity decrease was observed across all three solutions (Fig. S15). These alterations arise from the formation of tpy-Zn^2+^-tay or tpy-Zn^2+^-tey structures adopting characteristic sandwich-like geometries, which were stabilized by synergistic metal–ligand coordination and π–π stacking between anthracene/dimethoxylphenyl groups and the pyridine groups. Such stacking significantly intensifies nonradiative energy dissipation pathways, thereby inducing fluorescence decay. The titration profiles for the TAE + P5T + G2 mixture closely paralleled those of the P5T + 2 + 3 system (Fig. S15d and S16b). With increasing Zn^2+^ concentration, we observed a pronounced decrease in fluorescence emission intensity at 427 nm, accompanied by the emergence of a new emission band at 510 nm. This spectral transformation further supports the formation of both tpy-Zn^2+^-tay and tpy-Zn^2+^-tey in the TAE + P5T + G2 solution upon the addition of Zn^2+^.

**Fig. 3:**
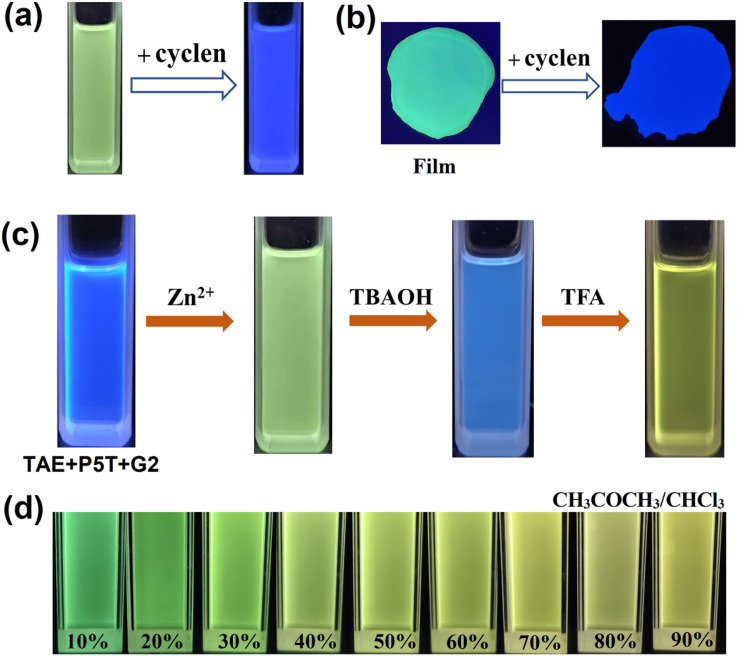
Photographs of the FSLP-based solution (a) and film (b) under 365 nm UV irradiation before and after the addition of cyclen, (c) the photographs of TAE + P5T + G2, TAE + P5T + G2 + Zn^2+^, TAE + P5T + G2 + Zn^2+^ + TBAOH, and TAE + P5T + G2 + Zn^2+^ + TBAOH + TFA under 365 nm UV irradiation, (d) fluorescence photographs of FSLP solutions at different mixed solvent under UV irradiation at 365 nm, where the acetone volume fraction ranges from 10% to 90%.

The responsiveness of FSLP to external stimuli was subsequently examined. As depicted in Fig. 3a, the FSLP solution or film containing TAE + P5T + G2 + Zn(OTf)_2_ emits chartreuse fluorescence when exposed to a 365 nm ultraviolet lamp. When cyclen,^43^ a strong chelating agent, was subsequently added to the FSLP solution or film containing TAE + P5T + G2 + Zn(OTf)_2_, a noticeable change occurred in the fluorescence emission, the color shifted from chartreuse to blue (Fig. 3a and b), accompanied by the emission enhancement. This fluorescence change can be explained as follows: cyclen forms a coordination complex with Zn^2+^, disrupting the tpy-Zn^2+^-tay and tpy-Zn^2+^-tey structures. Consequently, the coordinated tay, tey, and tpy groups revert to their free states, inducing the observed fluorescence emission changes.

Besides the chelating agent's ability to regulate the metal coordination, pH can also significantly impact metal coordination.^39^ After adding the organic base (TBAOH) to the FSLP solution, the fluorescence color of the solution changed from chartreuse to blue upon exposure to UV light (Fig. 3c). This observation implies that OH^−^ ions reacted with zinc ions, leading to the generation of insoluble Zn(OH)_2_ precipitate and the disruption of the tpy-Zn^2+^-tay and tpy-Zn^2+^-tey metal coordination structures. Consequently, the addition of TBAOH induced the coordinated ligands to become free ligands and caused the fluorescence changes of the solution. Interestingly, upon the subsequent addition of trifluoroacetic acid (TFA) to the solution, the fluorescence color of the solution was restored from blue to chartreuse (Fig. 3c). This is because trifluoroacetic acid can react with zinc hydroxide to reform zinc ions, which then coordinate with the ligands to reform coordination complexes. The pH-dependent reversible behavior demonstrates precise control over the metal coordination environment through acid–base regulation. The influence of solvent polarity on the fluorescence properties of supramolecular polymers was subsequently investigated. When the volume fraction of acetone in the mixed solvent system was incrementally increased from 10% to 90%, the fluorescence color of the FSLP solution progressively shifted from chartreuse to yellow (Fig. 3d). This observation demonstrates that variations in solvent polarity exert a substantial impact on the fluorescence emission characteristics of the FSLP solution.

**Fig. 4:**
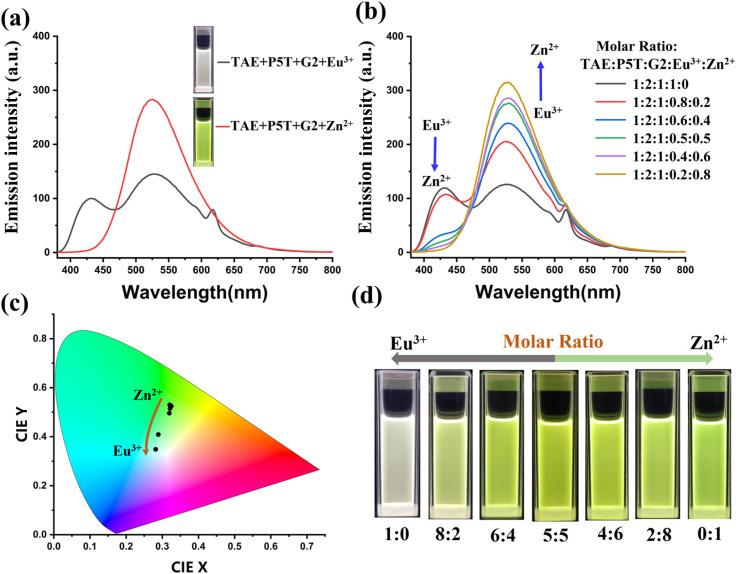
(a) Fluorescence emission of 0.02 mM TAE + P5T + G2 + Zn(OTf)_2_ and TAE + P5T + G2 + Eu(NO_3_)_3_ solutions (CHCl_3_/CH_3_COCH_3_ = 3 : 1, v/v), (b) fluorescence spectra of 0.02 mM TAE + P5T + G2 solutions upon adding different molar ratio of Zn(OTf)_2_ and Eu(NO_3_)_3_, (c) CIE chromaticity diagram of TAE + P5T + G2 + Zn(OTf)_2_ + Eu(NO_3_)_3_, (d) fluorescence photographs of TAE + P5T + G2 + Zn(OTf)_2_ + Eu(NO_3_)_3_ with different molar ratio of Zn^2+^/Eu^3+^.

Different metal ions or mixtures of metal ions can also modulate the structures and fluorescence emission of FSLP. Rare earth europium ions (Eu^3+^) have been shown to form metal coordination structures with certain ligands,^44^ such as terpyridine (tpy) ligands. In UV-vis titration experiments, Eu(NO_3_)_3_ was added to the solutions of model compounds 1, 1 + 2 (molar ratio 1 : 2 = 1 : 1), and 1 + 3 (molar ratio 1 : 3 = 1 : 1) to obtain titration curves. All absorption spectra of 1 + 2, 1 + 3, and 1 (Fig. S17a–c) displayed a peak near 285 nm, attributed to the π–π* transition. With increasing Eu^3+^ concentration, a new absorption peak emerged around 350 nm, indicating progressive coordination between the uncoordinated ligand and Eu^3+^. At a molar ratio of Eu^3+^/ligand = 1 : 2, maximum absorbance near 350 nm was observed, suggesting the formation of tpy-Eu^3+^-tpy, tpy-Eu^3+^-tay, and tpy-Eu^3+^-tey in the 1 + Eu^3+^, 1 + 2 + Eu^3+^, and 1 + 3 + Eu^3+^ solutions, respectively. This observation is consistent with ^1^H NMR analysis (Fig. S18 and S19). However, When Eu^3+^ was added to a solution of 2 + 3 (molar ratio 2 : 3 = 1 : 1), minimal changes were observed in the titration curve (Fig. S17d). This result suggests that the formation of tay-Eu^3+^-tay, tey-Eu^3+^-tey, and tay-Eu^3+^-tey structures is hindered by the large steric effects of dimethoxyphenyl and anthracyl groups. ^1^H NMR analysis further confirmed the steric hindrance imposed by these groups (Fig. S18e and S19e). Given the coordination abilities of Eu^3+^ and Zn^2+^ with these ligands, we investigated the fluorescence emission of FSLP by incorporating Zn^2+^ and Eu^3+^ into the solutions of TAE + P5T + G2 (molar ratio TAE : P5T : G2 = 1 : 2 : 1) at varying proportions. The mixture TAE + P5T + G2 + Eu^3+^ exhibited white fluorescence, while TAE + P5T + G2 + Zn^2+^ emitted yellow-green fluorescence (Fig. 4a). As the molar ratio of Zn^2+^/Eu^3+^ increased, the fluorescence color of TAE + P5T + G2 + Zn^2+^ + Eu^3+^ shifted from white to yellow-green (Fig. 4d). These results indicate that varying the Zn^2+^/Eu^3+^ molar ratio can significantly modulate the fluorescence emission of FSLP.

**Fig. 5:**
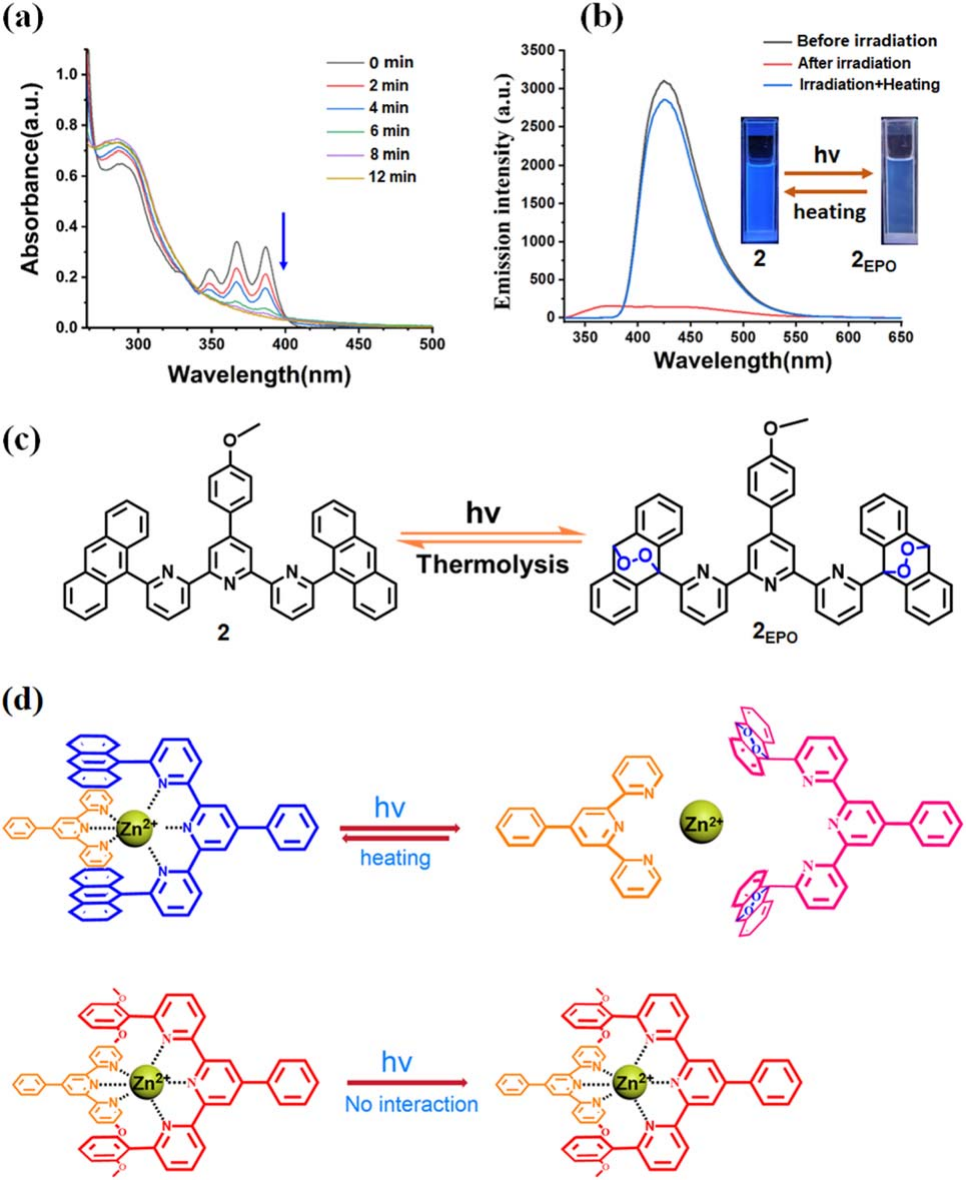
(a) Variation in the absorption spectra of model compound 2 ([2] = 0.02 mM) upon irradiation with a 365 nm UV lamp under an O_2_ atmosphere. (b) Fluorescence emission curves ([2] = 0.02 mM, *λ*_ex_ = 293 nm) under different conditions: before UV irradiation (black curve), after 12 min of irradiation at 365 nm (red curve), and after 12 min of irradiation at 365 nm followed by heating at 55 °C for 28 h under an N_2_ atmosphere (blue curve), along with images of model compound 2 before and after UV irradiation. (c) The chemical structures of 2_EPO_. (d) The depiction of the photoreactions of the 1 + 2 + Zn^2+^ and the 1 + 3 + Zn^2+^.

It is known that anthracenes possess photoresponsive properties.^45,46^ Anthracene can form dimers or transform into stable endoperoxides with singlet oxygen under UV light irradiation. It is prone to undergoing a photooxidation process when bulky substituent groups are attached to the 9,10-positions of anthracene, especially aryl-substituted groups.^47^ To investigate the photoresponsiveness of FSLP, we first studied the photoresponsiveness of model compound 2. When 2 was irradiated with 365 nm ultraviolet light for 12 minutes in an N_2_ atmosphere, the UV-vis absorption spectrum verified that the characteristic absorption peak of the anthracyl groups in the range of 340–400 nm on 2 remained unchanged (Fig. S20a), indicating that 2 cannot form a dimer due to the large substituent group at the 9-position of anthracene. However, when 2 was irradiated with 365 nm ultraviolet light for 12 minutes in an O_2_ atmosphere, the UV-vis absorption spectra revealed that the characteristic absorption band of the anthracyl groups in the range of 340–400 nm disappeared (Fig. 5a). The fluorescence intensity decayed strongly and the emission wavelength blueshifted from 425 nm to 380 nm (Fig. 5b). The change of fluorescence color also confirmed the interesting transformation of 2 under ultraviolet light irradiation in an O_2_ atmosphere. These observations indicate that the anthracene group on 2 undergoes a photo-oxygenation process with O_2_ to form its epoxide product 2_EPO_ under UV light irradiation. ^1^H NMR also supported the observation of photo-oxygenation, the proton A11 on the anthracene group showed a significant high-field shift (6.19 ppm, Fig. S21b). Furthermore, the formation of the 2_EPO_ product was directly verified by high-resolution ESI-MS at *m*/*z* = 756.2508, which corresponds to [2_EPO_ + H]^+^ (Fig. S22). Interestingly, heating 2_EPO_ at 55 °C for 28 h under an N_2_ atmosphere resulted in its almost complete reversion to the parent compound 2, which was verified by the fluorescence and ^1^H NMR spectra (Fig. 5b and S21c). Based on the above results, we verified that the anthracene group in model compound 2 can undergo reversible photooxygenation between anthracene and its oxygenated species (Fig. 5c). The monomer TAE exhibited a similar photo-oxygenation process to that of model compound 2, the anthracene group at the 6,6″-position of terpyridine underwent a photo-oxygenation process with O_2_ to form its epoxide product TAE_EPO_ under UV light irradiation (Fig. S23, S24b and S25), and TAE_EPO_ can transform back into the parent species TAE by heating at 55 °C for 28 h.

Subsequently, we investigated the photoresponsive property of the complementary metal coordination tpy-Zn^2+^-tay constructed by 1 + 2 + Zn^2+^. As expected, under an O_2_ atmosphere, when the 1 + 2 + Zn^2+^ was exposed to 365 nm ultraviolet irradiation for 20 minutes, the photo-oxygenation process was monitored *via* fluorescence spectroscopy. The fluorescence emission band exhibited a redshift from 425 nm to 450 nm, accompanied by an enhancement in fluorescence intensity (Fig. S26a). ^1^H NMR further furnished corroboration for the decomposition of tpy-Zn^2+^-tay. As shown in Fig. S27c, a complex ^1^H NMR spectrum was clearly observed upon irradiating the mixture of 1 + 2 + Zn^2+^, which appears to be a superposition of different coordination species. Furthermore, high-resolution ESI-MS provided more details regarding the degradation of the tpy-Zn^2+^-tay metal coordination under ultraviolet light in an O_2_ atmosphere. [Zn^2+^·1_2_] and [Zn^2+^·1·2_EPO_] were detected in the ESI-MS spectrum (Fig. S28). No [Zn^2+^·(2_EPO_)_2_] coordination species was detected, which may be due to the large steric hindrance of 2_EPO_. These experimental results indicate that the complementary tpy-Zn^2+^-tay metal coordination formed in the 1 + 2 + Zn^2+^ solution undergoes degradation to form endoperoxide 2_EPO_ after UV light irradiation under an O_2_ atmosphere (Fig. 5d). The resulting 2_EPO_, 1, and Zn^2+^ ions in solution can form a random mixture of [Zn^2+^·1_2_] and [Zn^2+^·1·2_EPO_] due to ligand exchange interactions. Interestingly, through the reversible transition between 2 and 2_EPO_, the ^1^H NMR signals of the assembly tpy-Zn^2+^-tay were reobserved after the solution of 1 + 2 + Zn^2+^ was illuminated for 20 minutes and then heated for 28 hours, indicating a reversible photo-oxygenation process by switching between light irradiation and heating (Fig. S27d). In contrast, the fluorescence spectrum of 1 + 3 + Zn(OTf)_2_ showed no change, implying that tpy-Zn^2+^-tey can remains stable under UV irradiation in an oxygen-containing atmosphere (Fig. S26b and 5d).

After determining the photo-oxidation behavior of 1 + 2 + Zn(OTf)_2_, we investigated the photoresponsive properties of FSLP constructed by TAE + P5T + G2 + Zn(OTf)_2_. When the mixed solution containing TAE + P5T + G2 + Zn(OTf)_2_ was exposed to UV irradiation in an O_2_ environment, the maximum emission peak blueshifted from 518 nm to 490 nm (Fig. S29e), along with an enhancement in intensity. In a parallel observation on the ^1^H NMR timescale (Fig. S30), irradiation of the same mixture led to a downfield shift for proton B5 and an upfield shift for the characteristic signal of proton B9. These results are in agreement with the earlier ^1^H NMR analysis from the UV irradiation experiment on model compounds 1 + 2 + Zn^2+^, suggesting that the tpy-Zn^2+^-tay coordination on the backbone of FSLP was destroyed due to the formation of the epoxide TAE_EPO_. FSLP underwent a disassembly process through the disruption of the sandwich-like tpy-Zn^2+^-tay coordination after UV irradiation. When the solution was subsequently heated, the fluorescence color reverted to the original color of TAE + P5T + G2 + Zn^2+^, and ^1^H NMR analysis also revealed the reformation of FSLP constructed from TAE + P5T + G2 + Zn^2+^ (Fig. S29e and S30c).

## Conclusions

In conclusion, we report the photo- and thermo-responsive regulation of terpyridyl-based complementary metal coordination, and demonstrate the construction of supramolecular polymers *via* dual complementary metal coordination and host–guest interactions. Under UV irradiation, the complementary metal coordination tpy-Zn^2+^-tay on the backbone of the FSLP underwent photooxidation, which triggered the destruction of the FSLP. Subsequent heating of the solution restored the tpy-Zn^2+^-tay coordination, thereby driving the reformation of the FSLP. Moreover, the FSLP exhibits tunable fluorescence emission by varying pH, solvent polarity, competitive ligands, and metal ion types. Given the fascinating coordination structures and attractive properties of supramolecular polymers, this study provides inspiration for designing new functional supramolecular assemblies.

## Author contributions

S. H. R. conceptualized the research and synthesized the monomers. Y. Z. W. and J.-A. Z. performed the polymer characterization studies. L. Q. L. and S. Y. L. analysed the data. L. L. L. provided suggestions during the study. Y. X., H. L. and W. T. conceived the experiment and reviewed the manuscript. All authors approved the final version of the manuscript.

## Conflicts of interest

The authors declare no conflicts of interest.

## Supplementary Material

SC-017-D6SC02381A-s001

## Data Availability

The data supporting this article have been included as part of the supplementary information (SI). Supplementary information: experimental methods, synthesis and characterization data for new compounds, and ESI-MS spectra. See DOI: https://doi.org/10.1039/d6sc02381a.
