# Supplementary material for: Photo- and thermo-responsive complementary metal coordination for the construction of supramolecular polymers
Source: Chem Sci. 2026 May 7;17(24):11962–72. doi: 10.1039/d6sc02381a (PMC13159016; doi:10.1039/d6sc02381a)
Supplement: SC-017-D6SC02381A-s001 [file SC-017-D6SC02381A-s001.pdf]

Supplementary Information (SI) for Chemical Science.
This journal is © The Royal Society of Chemistry 2026

Photo- and Thermo-Responsive Complementary Metal Coordination for the Construction of Supramolecular Polymers

Shenghui Rao,[a] Yuzhen Wang,[a] Ju-An Zhang,[b] Liqing Li,[a] Shaoyu Li,[a] Linlin Liu,[a] Yu Xie,[*c] Hui Li,[*a] Wei Tian[*b]

[a] Jiangxi Provincial Key Laboratory of Functional Crystalline Materials Chemistry, School of Chemistry and Chemical Engineering, Jiangxi University of Science and Technology, Ganzhou 341000, P. R. China.
[b] Shaanxi Key Laboratory of Macromolecular Science and Technology, School of Chemistry and Chemical Engineering, Northwestern Polytechnical University, Xi'an, 710072, P. R. China.
[c] College of Environment and Chemical Engineering, Nanchang Hangkong University, Nanchang 330063, P. R. China.
* E-mail: lh@jxust.edu.cn (H. Li.)
* E-mail: happytw_3000@nwpu.edu.cn (W. Tian)
* E-mail: xieyu_121@163.com (Y. Xie)

Supporting information

1. Materials and methods……………………………………………………………...1
2. Self-sorting binding investigation based on model compounds 1-6………………..2
3. 2D COSY NMR spectra of TAE+P5T+G2+$Zn(OTf)_2$……………………………..6
4. Concentration-dependent ^{1}H NMR spectra…………………………......................7
5. UV-Vis titration experiments by adding Zn^{2+} ions………………………………8
6. 2D DOSY NMR spectrum……………………………………………………………9
7. TEM image……………………………………………………………………………9
8. The calculated value of maximum degree of polymerization n_{max}………………...11
9. Fluorescence titration experiments………………………………......................11
10. UV-Vis absorbance by adding Eu^{3+}………………………………………………13
11. ^{1}H NMR analysis of model compounds by adding Eu^{3+}…………………………13
12. The study of photoresponsiveness…………………………………………………..14
13. Stability investigation of host-guest complexes after UV irradiation…………….21
14. The discussion of binding constants…………………………………………………22
15. Synthesis of intermediates and monomers………………………………………..24

1. Materials and Methods

The reagents and solvents were used directly as purchased. Column chromatography was conducted on silica gel (200-300 mesh). All reactions were performed under atmospheric conditions unless otherwise specified. ^{1}H NMR and ^{13}C NMR spectra were carried out on a Bruker DPX 400 MHz spectrometer. High-resolution electrospray ionization mass spectra (HR-ESI-MS) were obtained on a Bruker Esquire 3000 plus mass spectrometer equipped with an ESI interface and ion trap analyzer. Viscosity measurements were carried out with Ubbelohde micro viscometers (Shanghai Liangjing Glass Instrument Factory, 0.40 mm inner diameter) at 298 K in chloroform/ acetone (3/1, v/v). The UV/vis absorption experiment was conducted on a PerkinElmer L950 device. Fluorescence spectroscopy was performed on a HZTACHZ F-2500 spectrometer, the slit widths were 10 nm for excitation and emission.

FSLP was prepared by mixing TAE, P5T, G2, and $Zn(OTf)_2$ in a 1:2:1:2 molar ratio, followed by dissolution in $CDCl_3/CD_3COCD_3$ (3:1, v/v). The resulting mixture was heated to 50 °C and subsequently cooled to room temperature to yield the target supramolecular polymer FSLP.

TEM samples of FSLP constructed by TAE+P5T+G2+$Zn(OTf)_2$ were prepared at a concentration of 35 mM. A drop of the sample solution (chloroform/acetone = 3/1, v/v) was deposited onto a carbon-coated copper grid. After solvent evaporation, TEM images were recorded using a Tecnai-G2 instrument.

Photooxidation experiments were conducted using a photochemical reaction system equipped with a 500 W Hg lamp and an optical filter to generate UV light centered at 365 nm. The epoxide products 2_{EPO} and TAE_{EPO} were synthesized by irradiating compound 2 and TAE with 365 nm UV light under an oxygen atmosphere for 12 minutes. The deoxygenation of 2_{EPO} and TAE_{EPO} was carried out by heating at 55 °C for 28 h under an N_2 atmosphere.

2. Self-sorting binding investigation based on model compounds 1-6

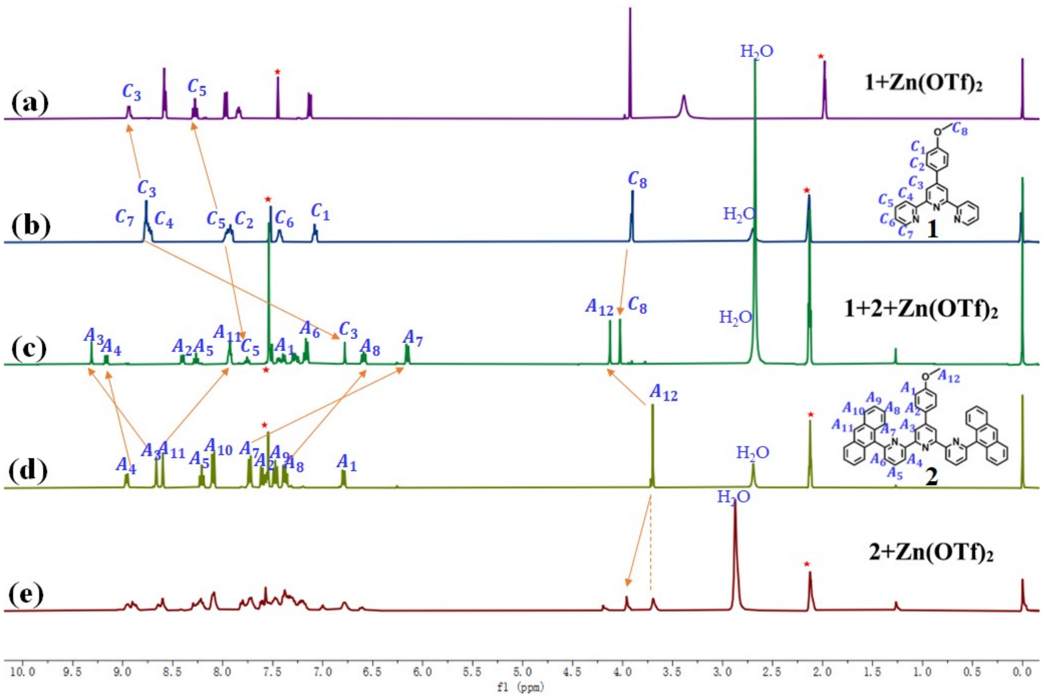

Fig. S1 ^{1}H NMR spectra (400 MHz, $CDCl_3/CD_3COCD_3$= 3/1, *v*/*v*) of (a) 1+Zn(OTf)$_2$, (b) 1, (c) 1+2+Zn(OTf)$_2$, (d) 2, (e) 2+Zn(OTf)$_2$.

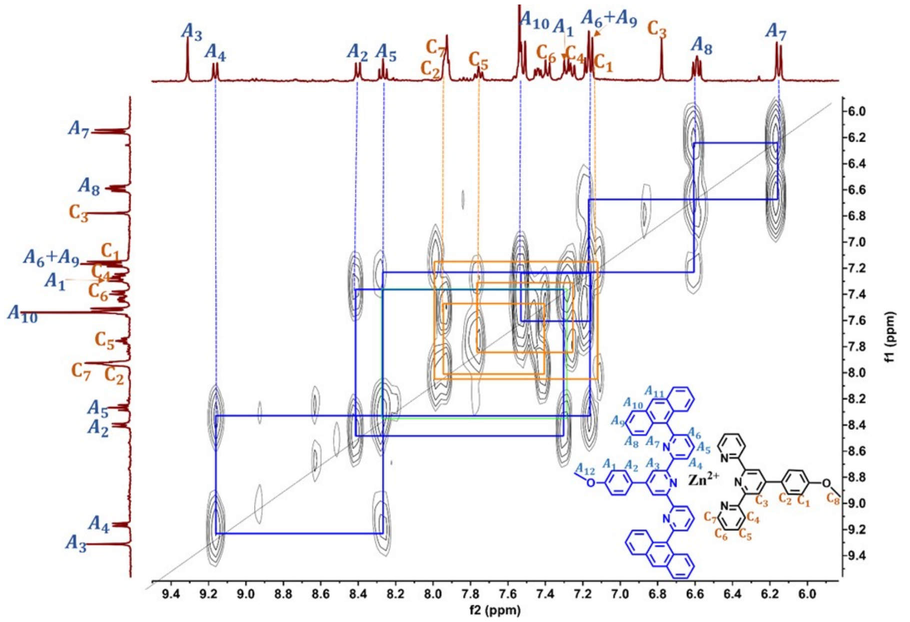

Fig. S2 2D ^{1}H-^{1}H COSY NMR (600 MHz, 40 mM) spectra of 1+2+Zn(OTf)$_2$ in $CDCl_3$-CD_3COCD_3 (3/1, *v*/*v*).

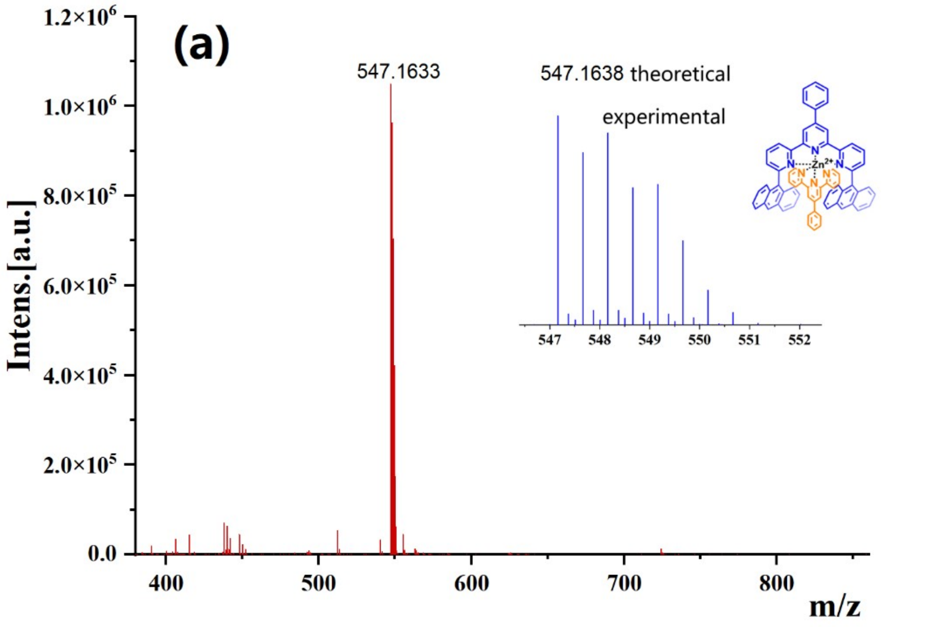

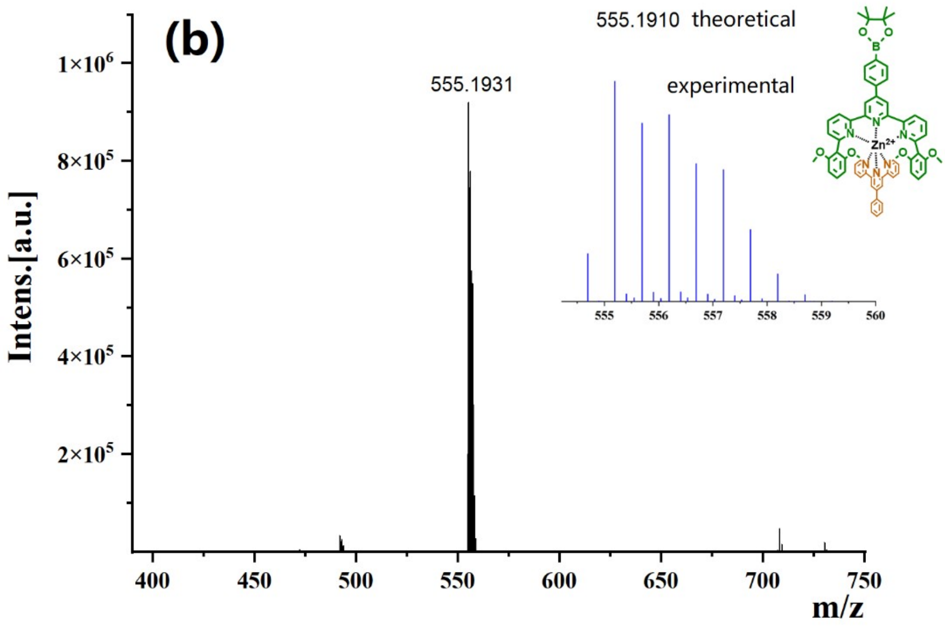

Fig. S3 High-resolution ESI-MS mass spectra of (a) 1+2+$Zn(OTf)_2$ and (b) 1+3+$Zn(OTf)_2$.

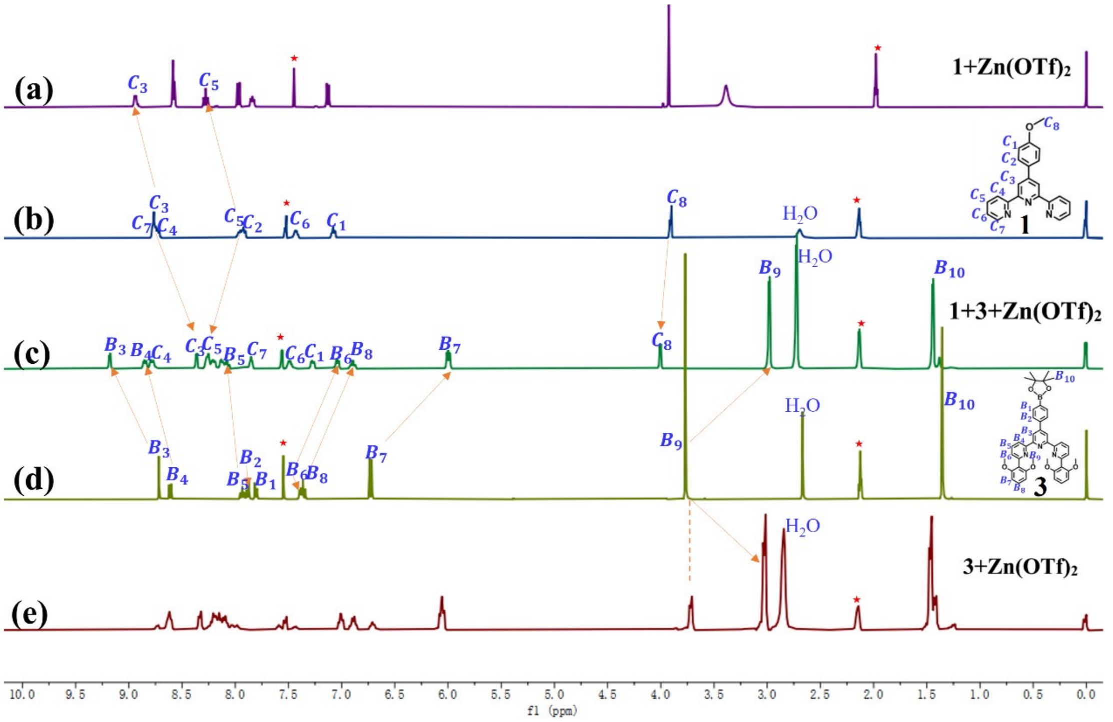

Fig. S4 ^{1}H NMR spectra (400 MHz, $CDCl_3/CD_3COCD_3$= 3/1, *v*/*v*) of (a) 1+$Zn(OTf)_2$, (b) 1**,** (c) 1+3+$Zn(OTf)_2$, (d) 3, (e) 3+$Zn(OTf)_2$.

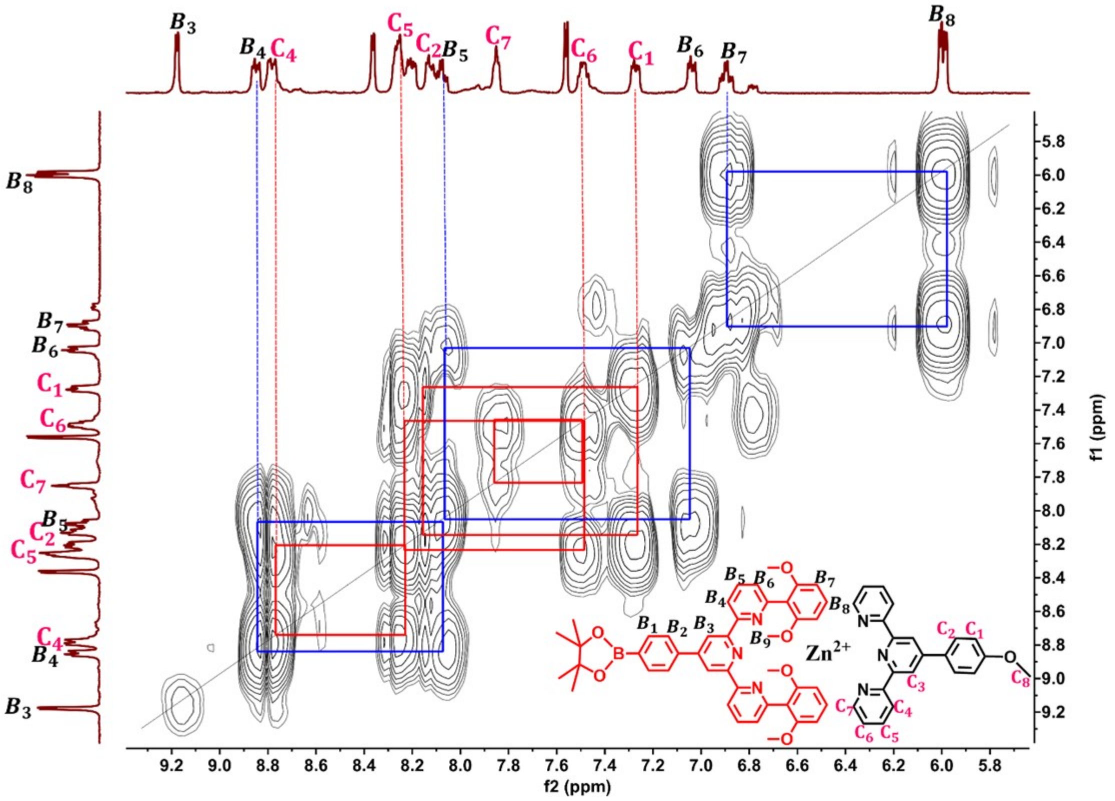

Fig. S5 2D ^{1}H-^{1}H COSY NMR (400 MHz, 40 mM) spectra of 1+3+$Zn(OTf)_2$ in $CDCl_3$-CD_3COCD_3 (3/1, *v*/*v*).

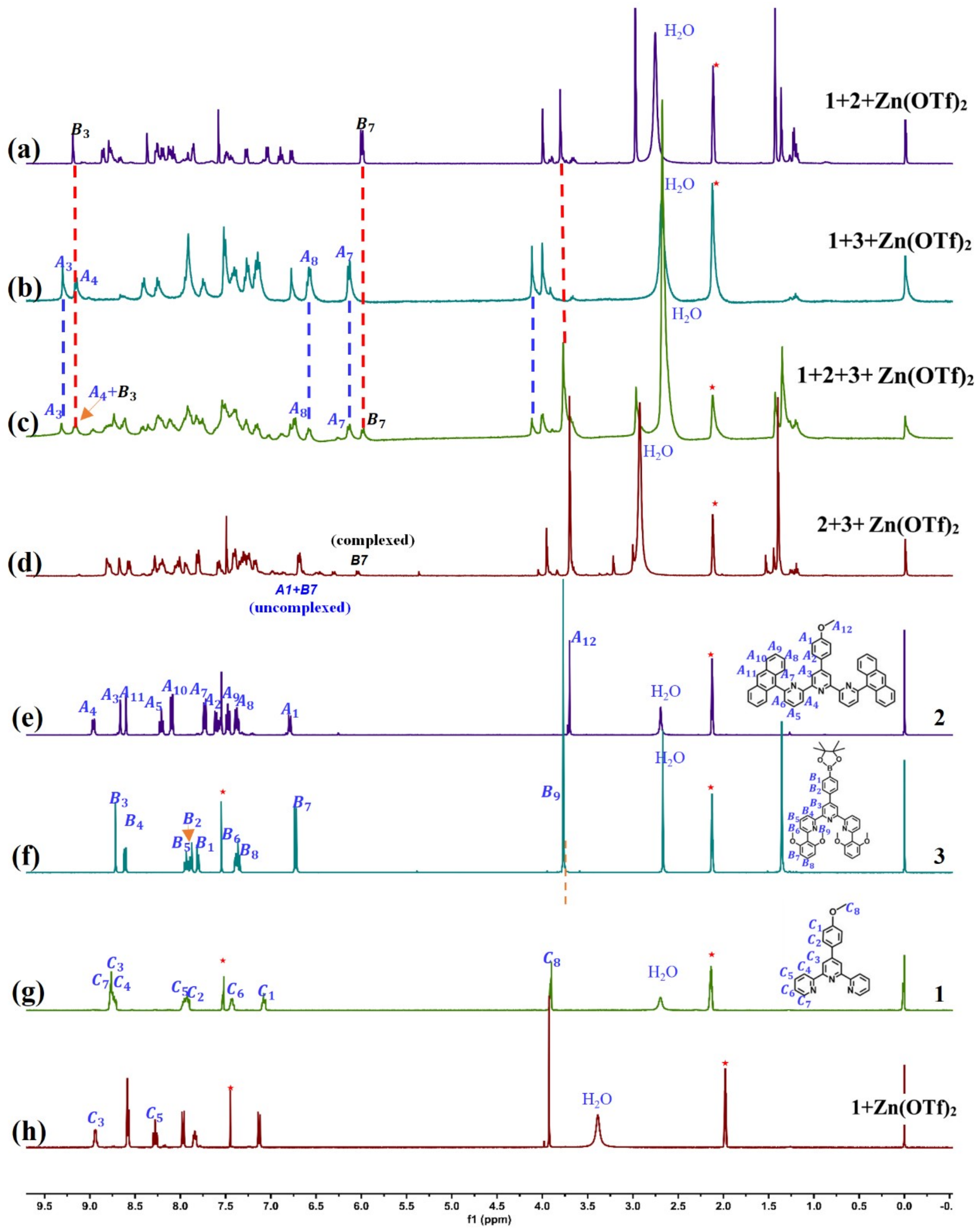

Fig. S6 ^{1}H NMR spectra (400 MHz, $CDCl_3/CD_3COCD_3$= 3/1, *v*/*v*) of (a) 1+2+Zn$(OTf)_2$(molar ratio: 1:2:Zn$(OTf)_2$=1:1:1) (b) 1+3+Zn$(OTf)_2$ (molar ratio: 1:3:Zn$(OTf)_2$=1:1:1), (c) 1+2+3+Zn$(OTf)_2$ (molar ratio: 1:2:3:Zn$(OTf)_2$=2:1:1:2), (d) 2+3+Zn$(OTf)_2$ (molar ratio: 2:3:Zn$(OTf)_2$=1:1:1), (e) 2, (f) 3, (g) 1, (h) 1+Zn$(OTf)_2$ (molar ratio: 1:Zn$(OTf)_2$=2:1).

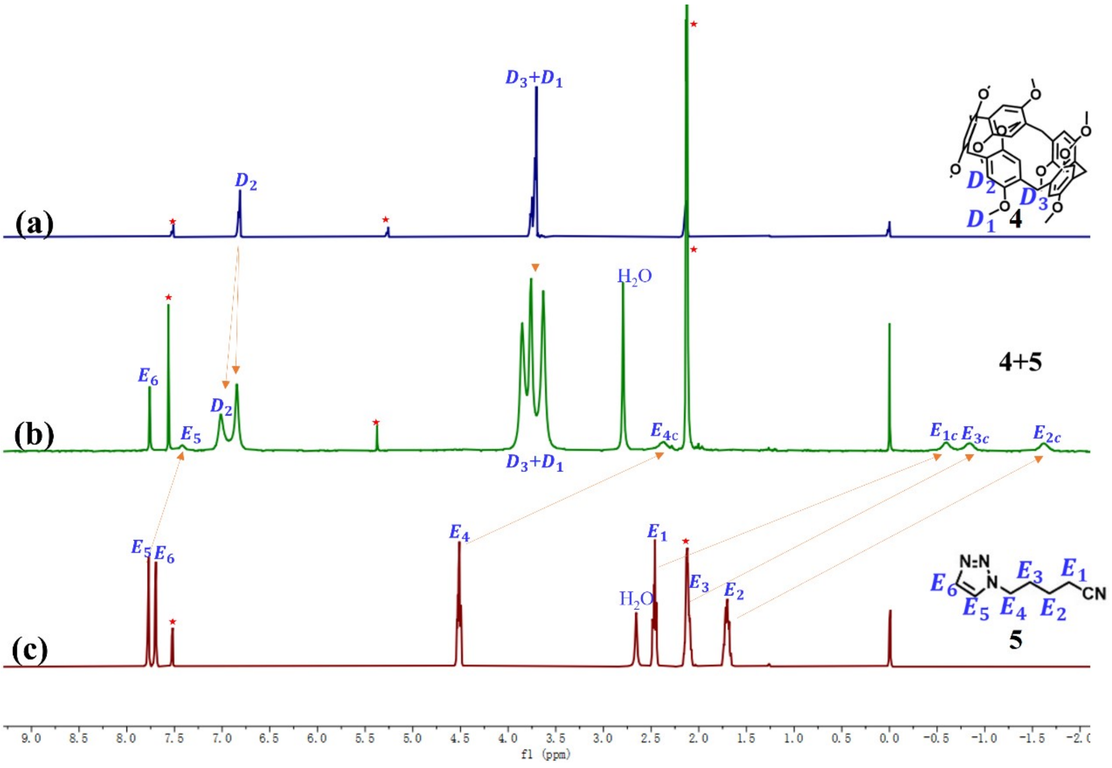

Fig. S7 ^{1}H NMR spectra (400 MHz, $CDCl_3/CD_3COCD_3$= 3/1, *v/v*) of (a) 4, (b) 4+5, (c) 5.

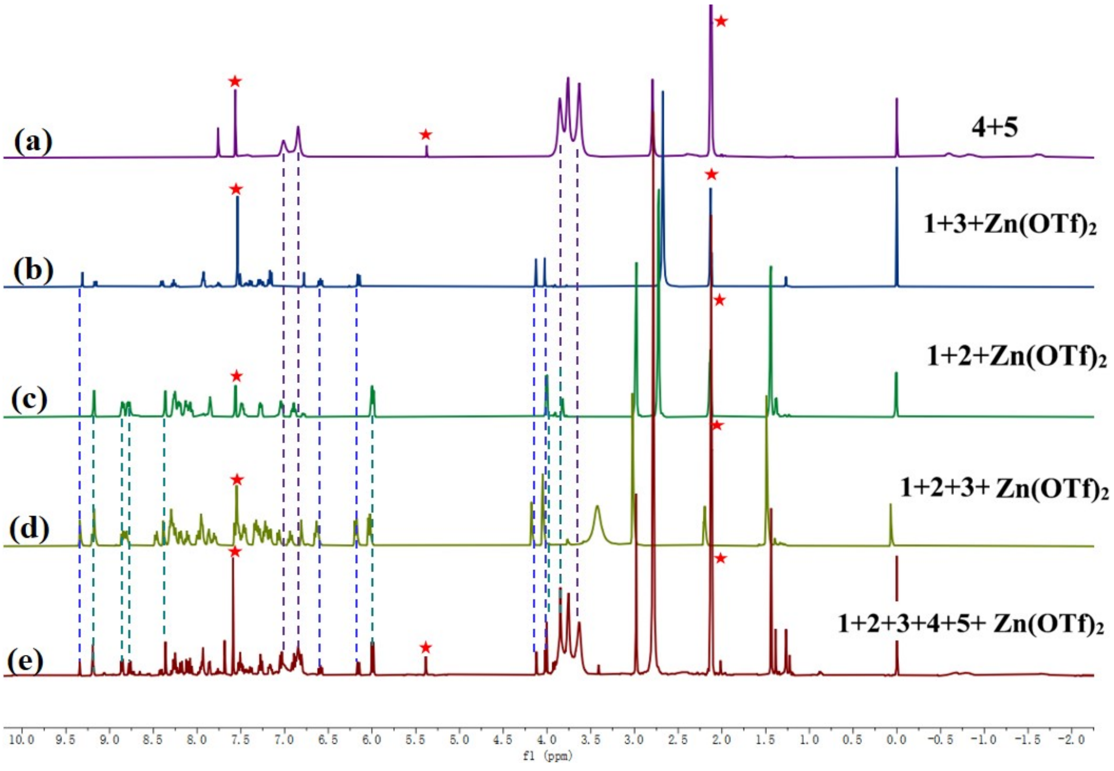

Fig. S8 ^{1}H NMR spectra (400 MHz, $CDCl_3/CD_3COCD_3$= 3/1, *v/v*) of (a) 4+5, (b) 1+3+$Zn(OTf)_2$, (c) 1+2+$Zn(OTf)_2$, (d) 1+2+3+$Zn(OTf)_2$, (e) 1+2+3+4+5+$Zn(OTf)_2$.

3. 2D COSY NMR and NOESY spectra of TAE+P5T+G2+Zn(OTf)$_2$

(a)

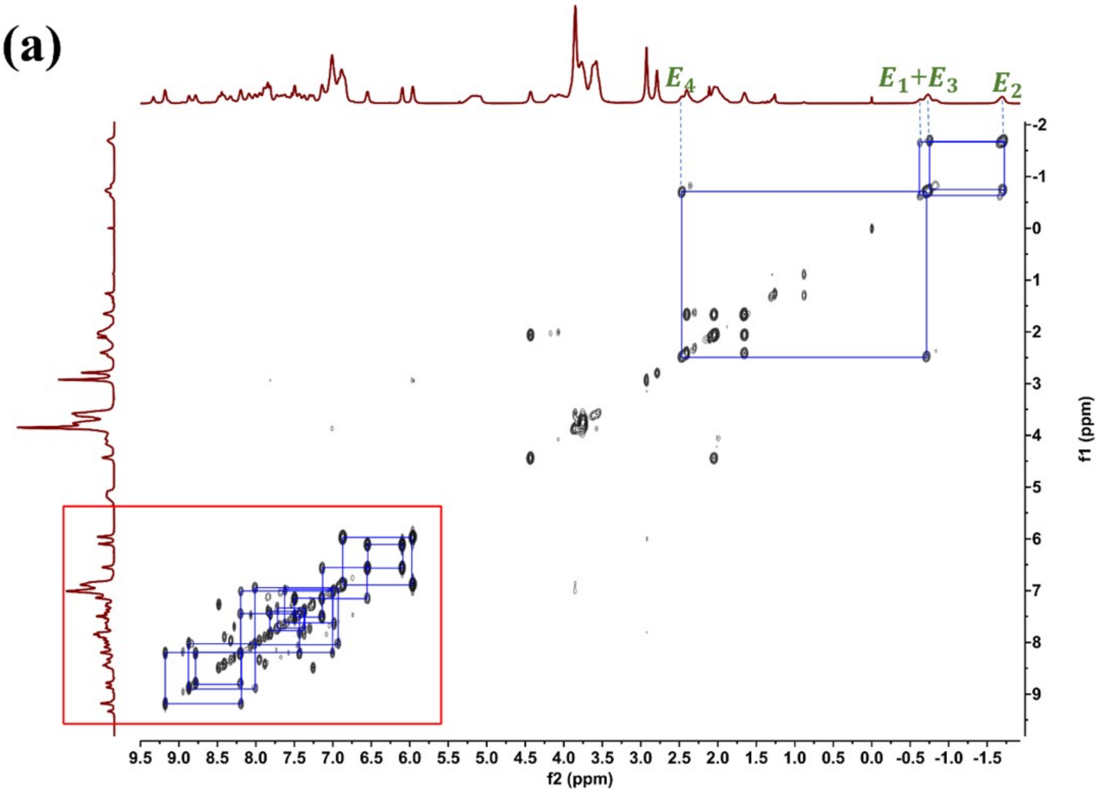

(b)

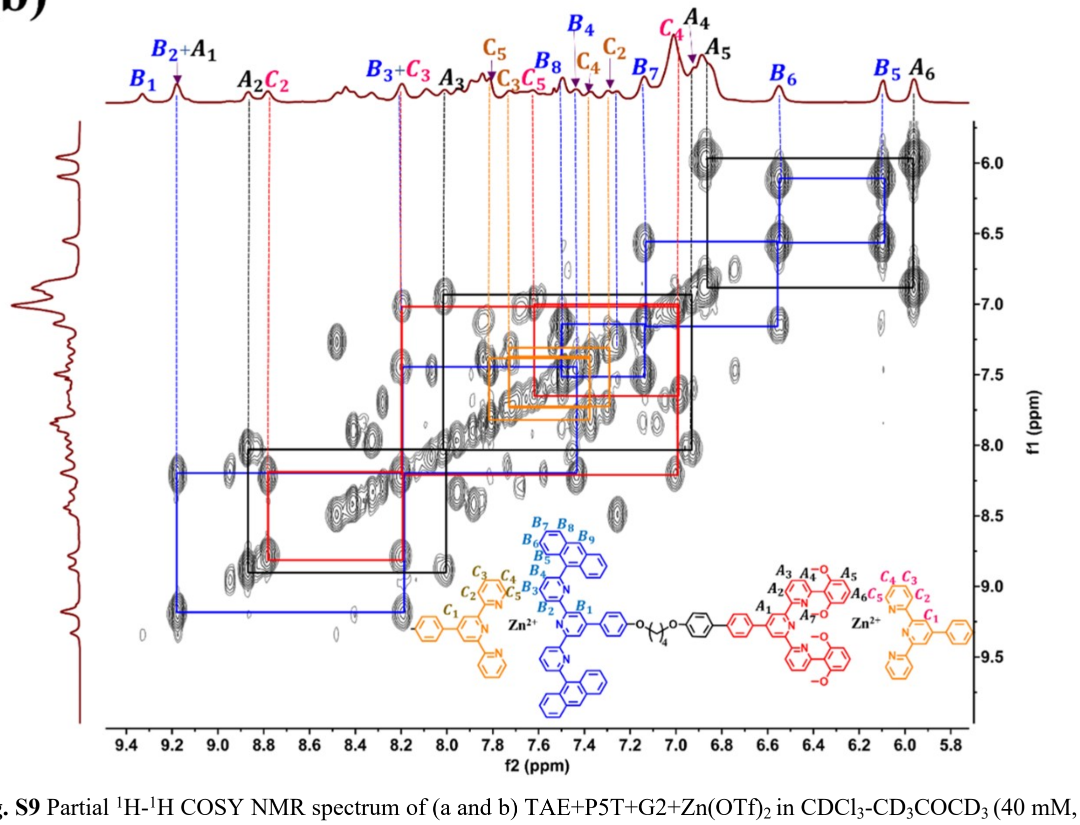

Fig. S9 Partial ^{1}H-^{1}H COSY NMR spectrum of (a and b) TAE+P5T+G2+Zn(OTf)$_2$ in $CDCl_3$-CD_3COCD_3 (40 mM, 3/1, *v*/*v*).

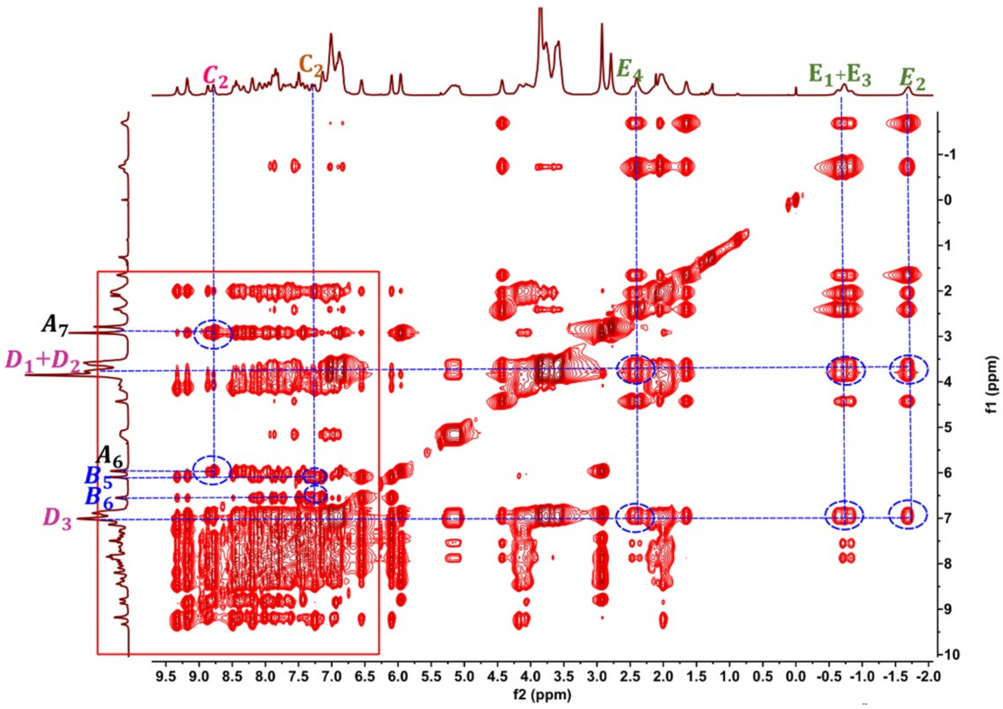

Fig. S10 The NOESY NMR of TAE+P5T+G2+Zn(OTf)$_2$ in $CDCl_3/CD_3COCD_3$ (v/v = 3/1, 40 mM, molar ratio: TAE: P5T: G2: Zn(OTf)$_2$ = 1:2:1:2).

4. Concentration-dependent ^{1}H NMR spectra

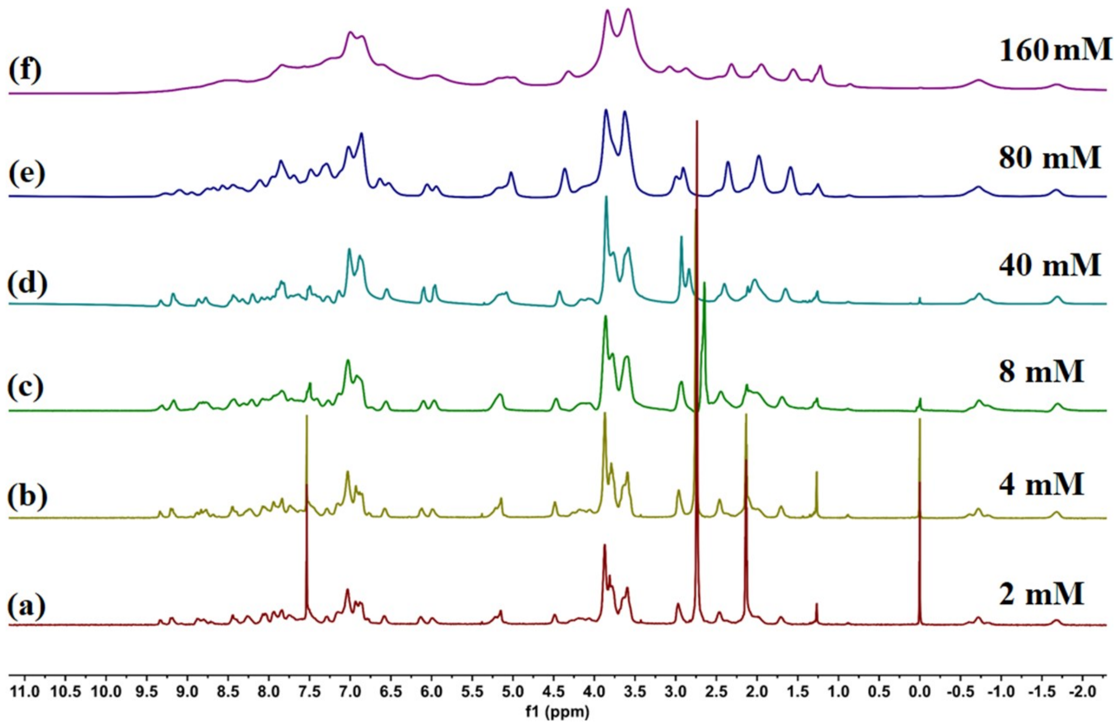

Fig. S11 ^{1}H NMR spectra (400 MHz, $CDCl_3/CD_3COCD_3$= 3/1, *v*/*v*) of TAE+P5T+G2+Zn(OTf)$_2$ (molar ratio: 1:2:1:2) at different concentrations of TAE (a) 2 mM, (b) 4 mM, (c) 8 mM, (d) 40 mM, (e) 80 mM, (f) 160 mM.

5. UV-Vis titration experiments by adding Zn^{2+} ions

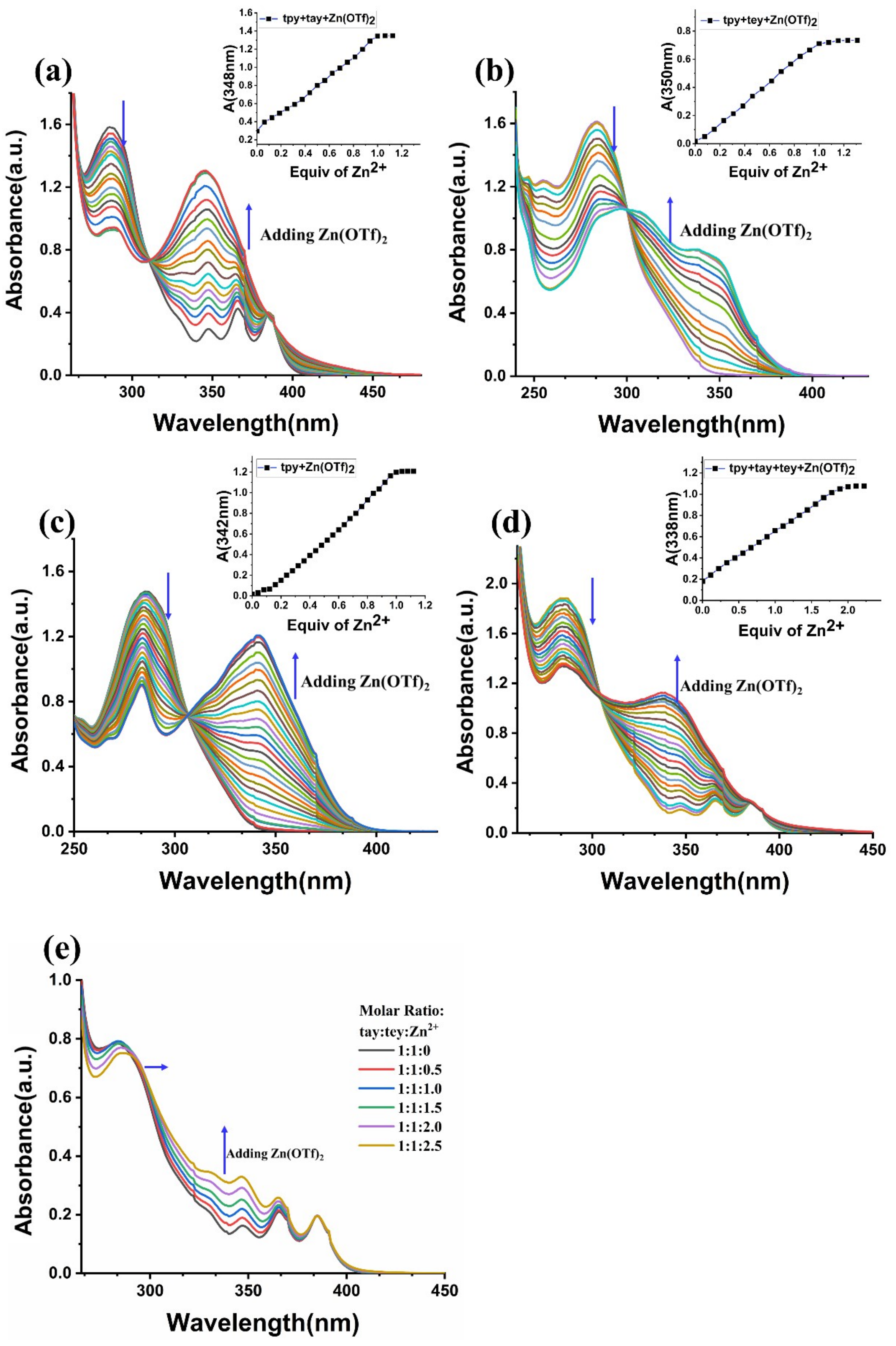

Fig. S12 The changes of the UV-Vis absorbance through the stepwise addition of $Zn(OTf)_2$ into a 0.02 mM solution of (a) 1+2, (b) 1+3, (c) 1, (d) 1+2+3, (e) 2+3.

6. 2D DOSY NMR spectrum

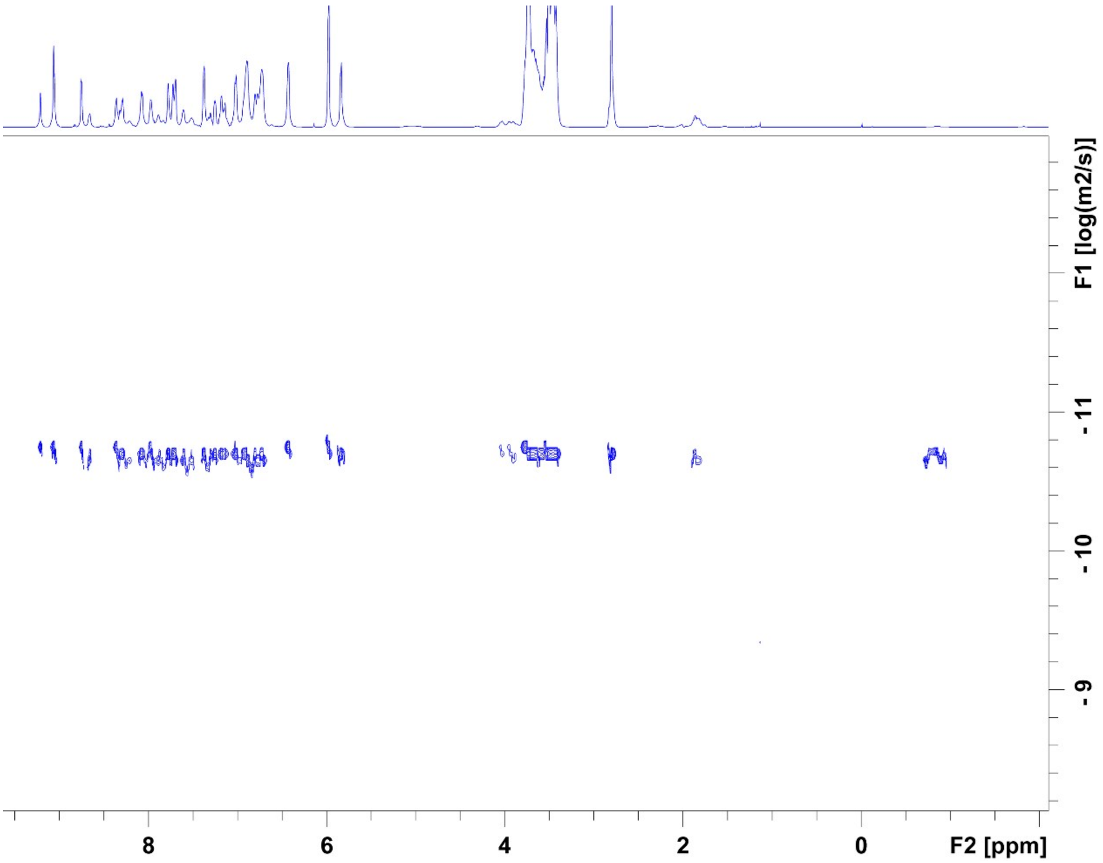

Fig. S13 Representative DOSY spectrum (600 MHz, $CDCl_3/CD_3COCD_3$= 3/1, *v*/*v*) of the solution of TAE+P5T+G2+$Zn(OTf)_2$ at a concentration of 50 mM (molar ratio: TAE:P5T:G2:$Zn(OTf)_2$=1:2:1:2).

7. TEM image

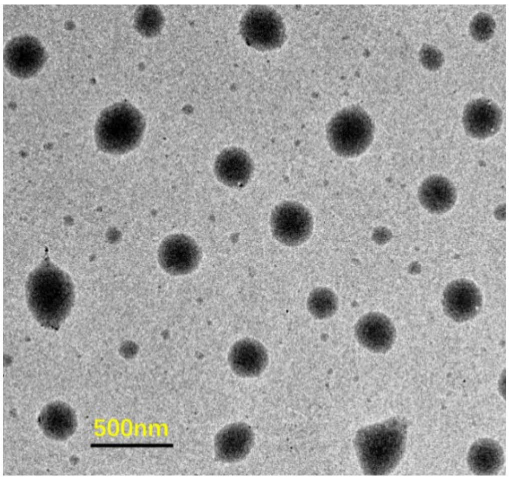

Fig. S14 Representative TEM image of TAE+P5T+G2+$Zn(OTf)_2$.

8. The calculated value of maximum degree of polymerization n_{max}.

For the TAE+G2+P5T+$Zn(OTf)_2$ system, the maximum possible degree of polymerization (n_{max}) could be estimated using a reported method by Gibson and coworkers.[S1] Using this method and assuming an same average association constant for each successive binding step (isodesmic model), the maximum possible degree of polymerization(n_{max}) is correlated with the equilibrium constant K and the initial monomer concentration. It can be easily deduced as follows:

$$n_{max} = (2K\,[Host]_0)^{1/2},$$

where $[Host]_0 = [P5T]$.[S1]

The degree of polymerization (n_{max}) for TAE+G2+P5T+$Zn(OTf)_2$ system at a concentration of 80 mM (80 mM P5T + 40 mM TAE + 40mM G2+ 80mM $Zn(OTf)_2$) was calculated to be 39.8, with the hypothesis that the P5T•Zn^{2+}•TAE•Zn^{2+}•P5T dissociation was negligible in the solution because the association constants of tpy-Zn^{2+}-tay and tpy-Zn^{2+}-tey are much larger than that of P5-TPN. Thus, the repeat units of in the copolymer $[P5T•Zn^{2+}•TAE•Zn^{2+}•P5T•G2]_n$ are about 39.8, and the molecular weight of supramolecular polymer FSLP is about 39.8*(4351 Da)=173.2 kDa.

9. Fluorescence titration experiments

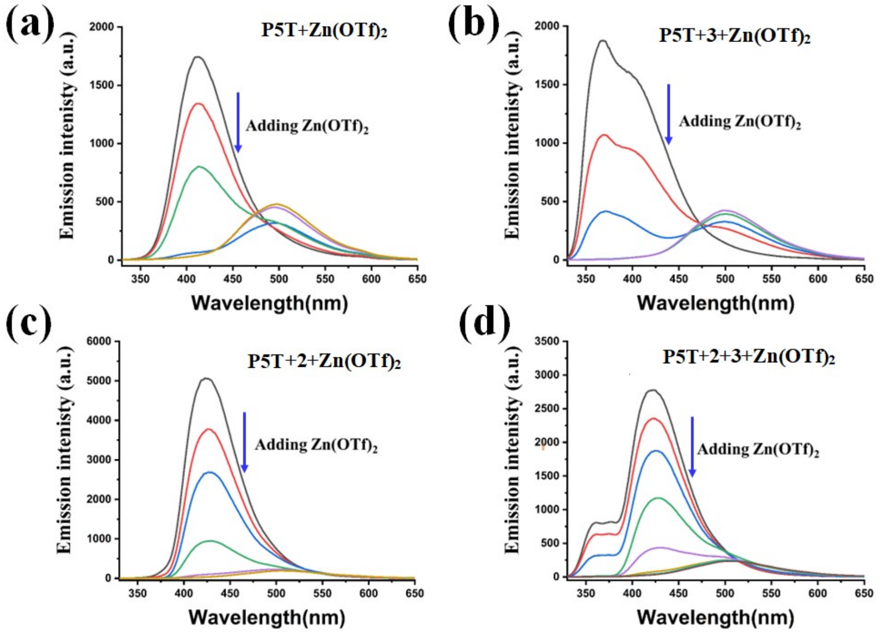

Fig. S15 The changes of fluorescence emission by the dropwise addition of $Zn(OTf)_2$ into a 0.02 mM solution ($CHCl_3/CH_3COCH_3$=3/1, v/v) of (a) P5T, (b) P5T+3, (c) P5T+2, (d) P5T+2+3.

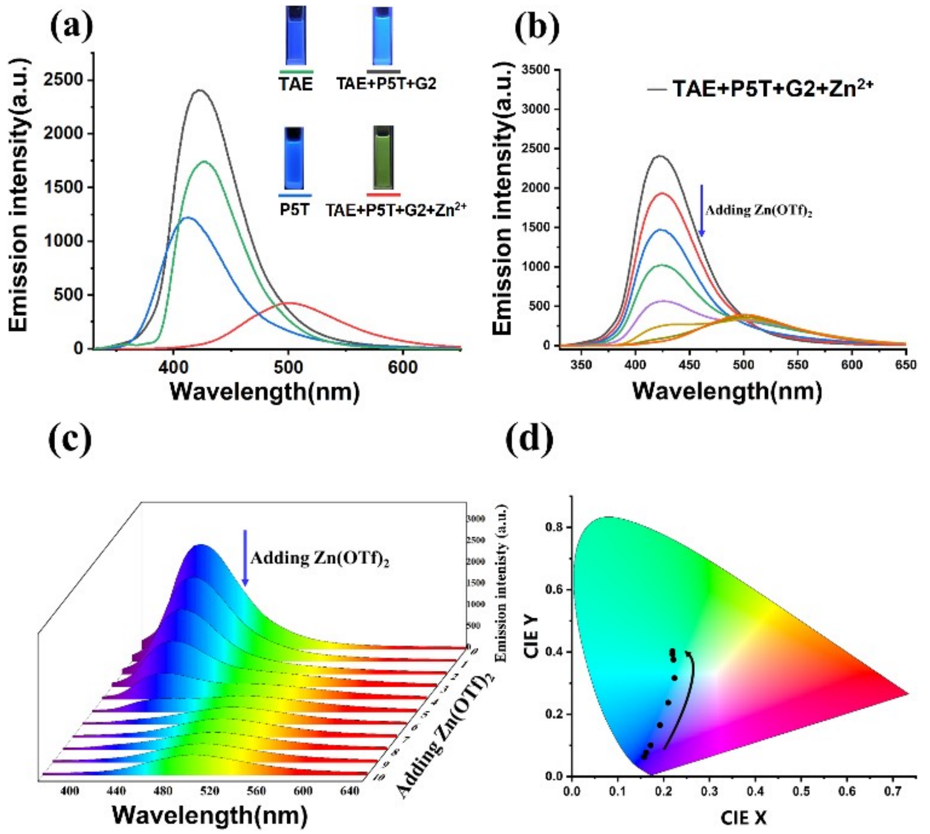

Fig. S16 (a) The fluorescence emission curves of a 0.02 mM solution ($CHCl_3/CH_3COCH_3$=3/1, v/v) of TAE, P5T, TAE+P5T+G2 and TAE+P5T+G2+Zn(OTf)$_2$, λ_{ex}=293 nm inset: the photographs of fluorescence emission, (b-c) the evolution of fluorescence upon dropwise addition of Zn(OTf)$_2$ to a 0.02 mM solution of TAE+P5T+G2, λ_{ex}=293 nm, (d) the CIE chromaticity diagram of the TAE+P5T+G2 upon stepwise addition of Zn(OTf)$_2$.

10. UV-Vis absorbance by adding Eu^{3+}

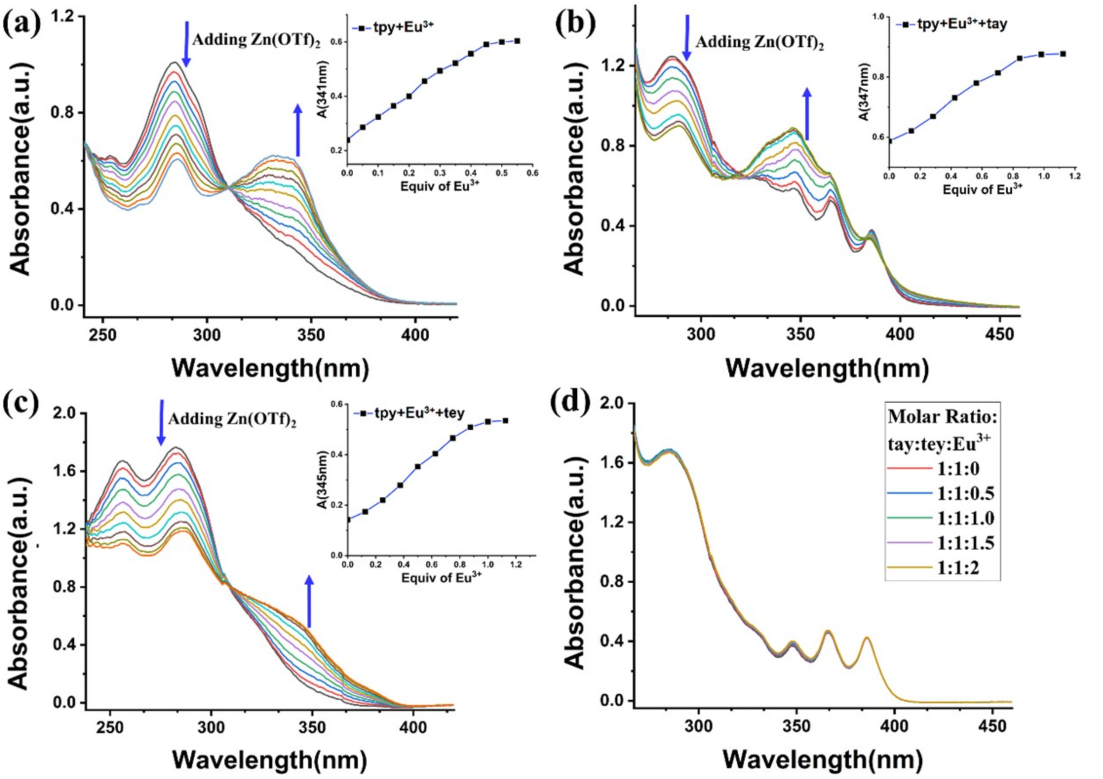

Fig. S17 The changes of the UV-Vis absorbance through the stepwise addition of $Eu(NO_3)_3$ into a 0.02 mM solution of (a) 1, (b) 1+2, (c) 1+3, (d) 2+3.

11. ^1H NMR analysis of model compounds by adding Eu^{3+}

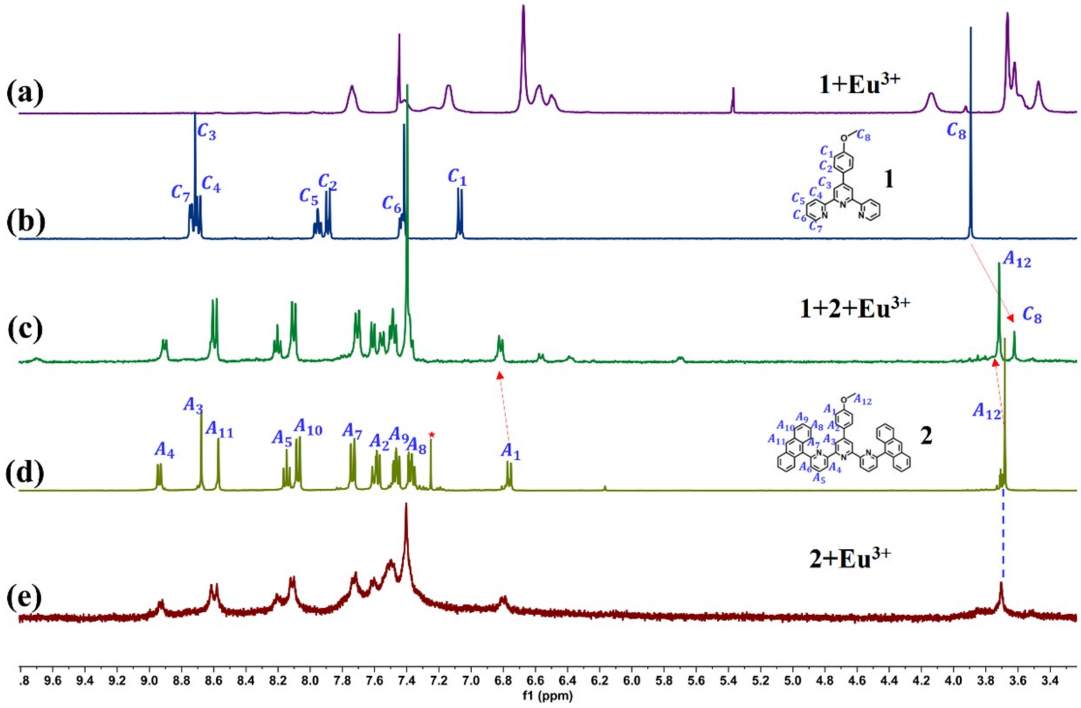

Fig. S18 ^{1}H NMR spectra (400 MHz, $CDCl_3/CD_3COCD_3$= 3/1, *v*/*v*) of (a) 1+Eu$(NO_3)_3$, (b) 1, (c) 1+2+ Eu$(NO_3)_3$, (d) 2, (e) 2+Eu$(NO_3)_3$.

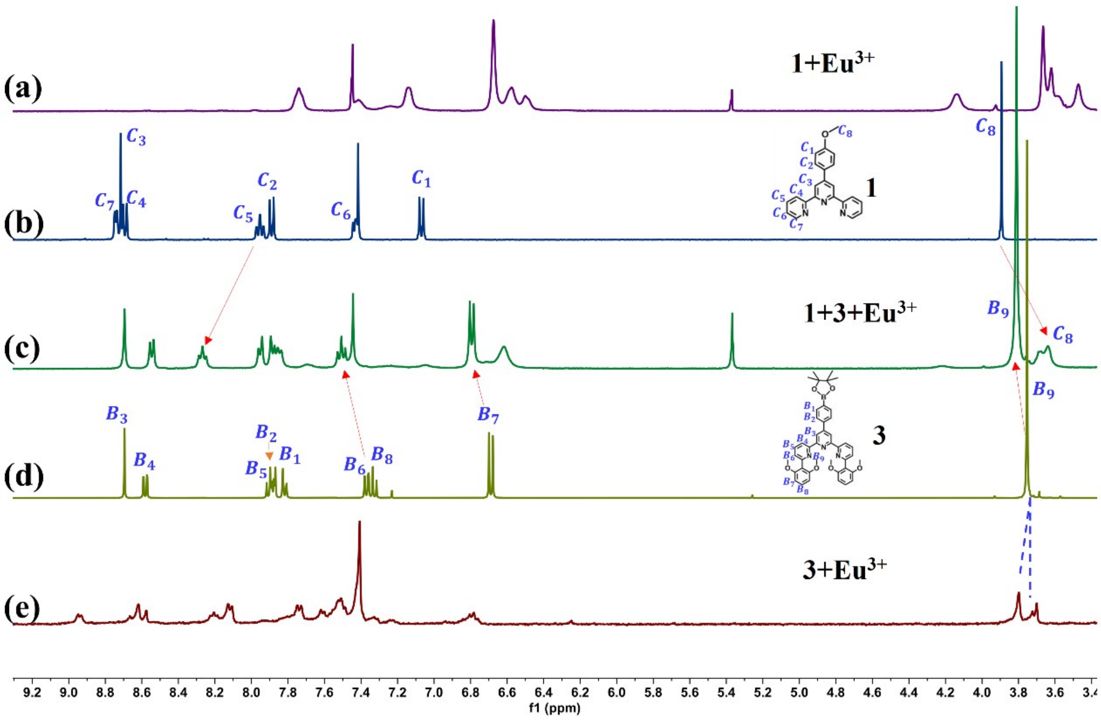

Fig. S19 ^{1}H NMR spectra (400 MHz, $CDCl_3/CD_3COCD_3$= 3/1, *v*/*v*) of (a) 1+ Eu$(NO_3)_3$, (b) 1, (c) 1+3+ Eu$(NO_3)_3$, (d) 3, (e) 3+Eu$(NO_3)_3$.

12. The study of photoresponsiveness

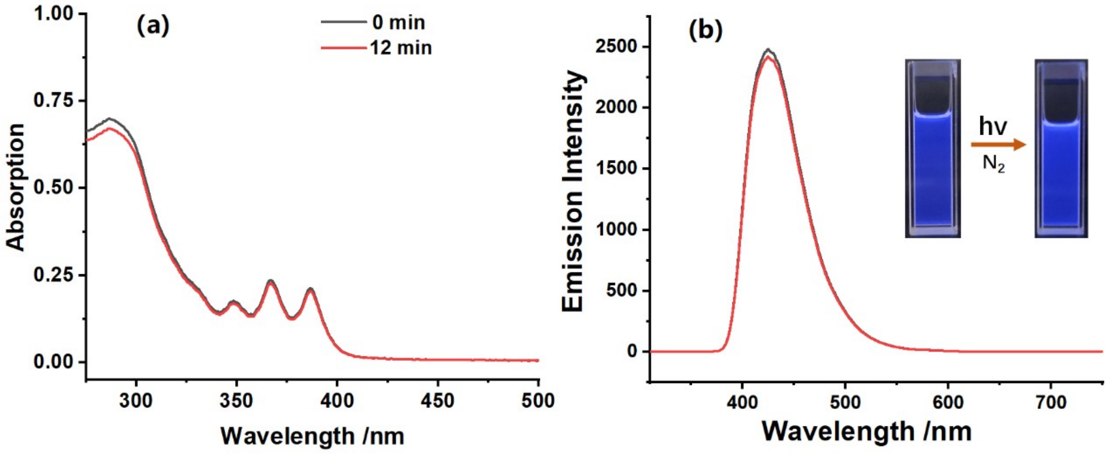

Fig. S20 (a) Absorption spectra variation of the model compound 2 ([2] = 0.02 mM) upon irradiation by UV lamp at 365 nm under an N_2 atmosphere. (b) The fluorescence curves ([2] = 0.02 mM, λ_{ex} = 365 nm) and the images of the model compound 2 before and after photooxidation.

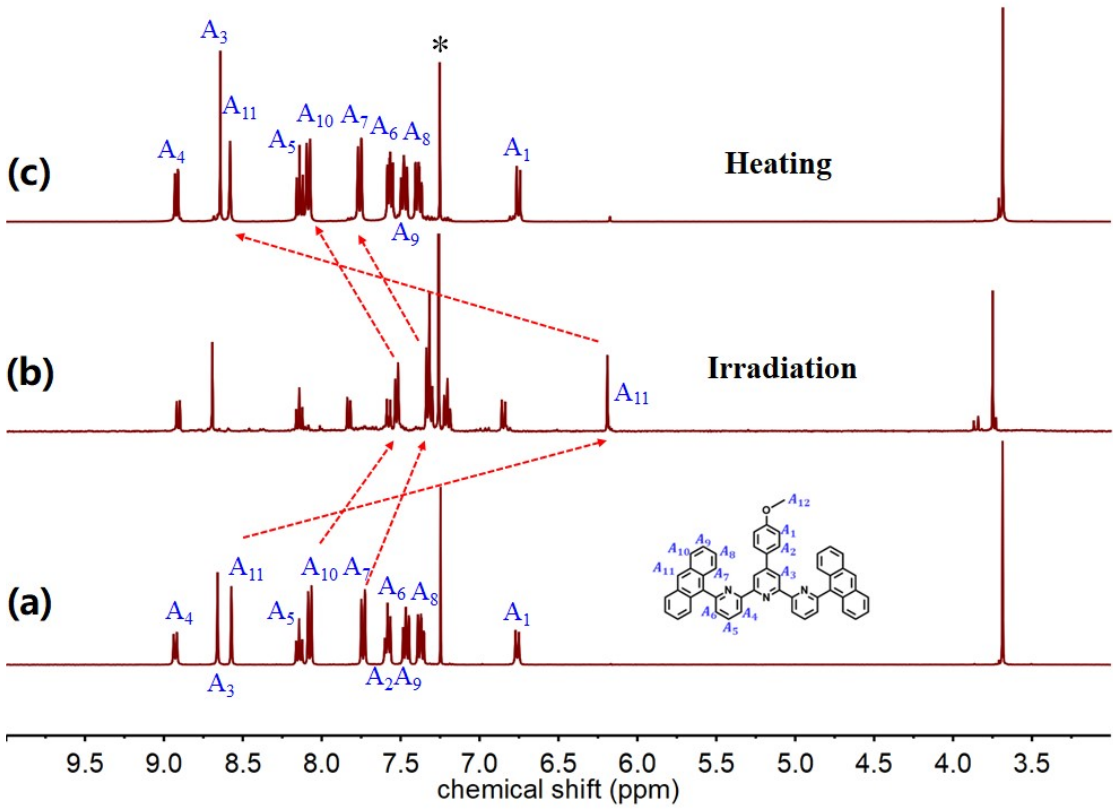

Fig. S21 ^{1}H NMR spectra (400 MHz, $CDCl_3$, 298 K) of the reversible photo-oxygenation of model compound 2 ([2] = 10 mM) under an O_2 atmosphere: (a) before irradiation with UV light, (b) after irradiation 12 min at 365 nm, (c) after irradiation at 365 nm for 12 min and heating at 55 °C for 28 h.

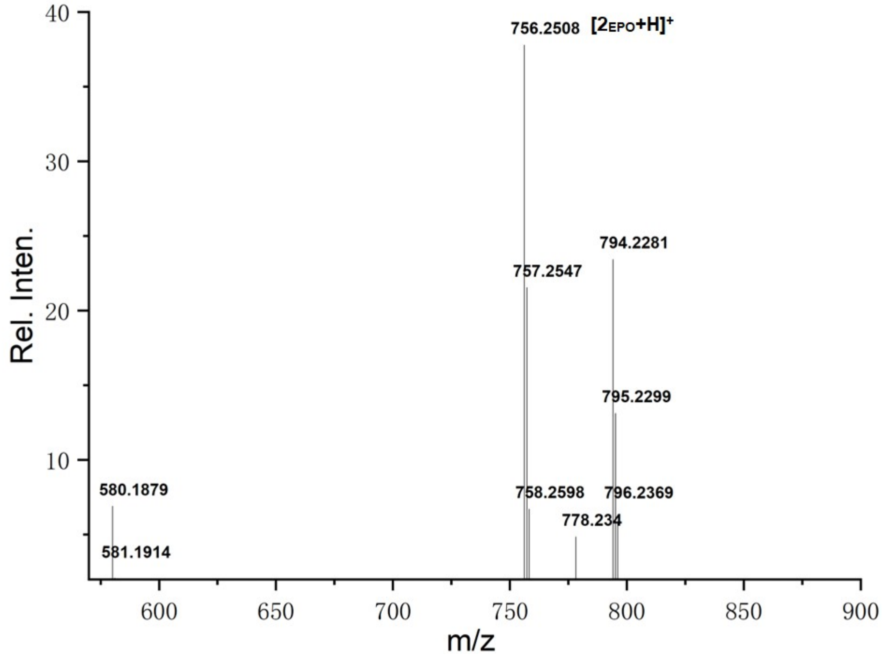

Fig. S22 High-resolution HR-ESI-MS mass spectrum of compound $\mathbf{2}_{EPO}$. m/z calcd for $[2_{EPO}+H]^+$=756.2493, found=756.2508, error=1.98 ppm.

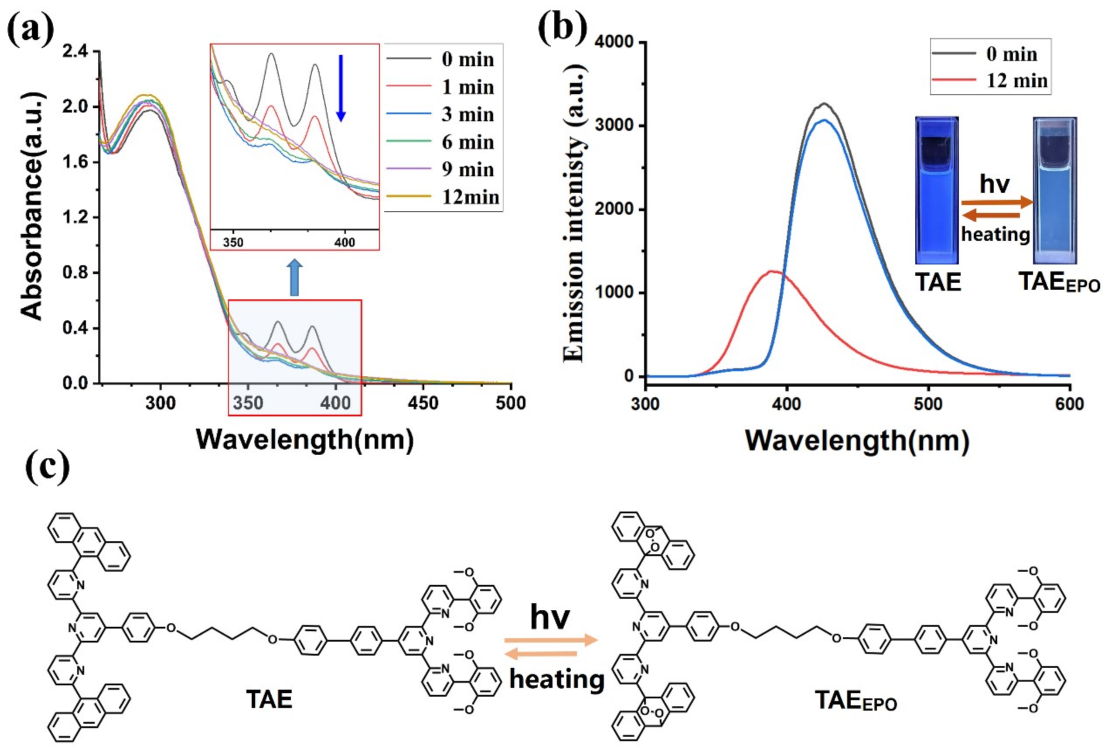

Fig. S23 (a) Absorption spectra variation of TAE (0.02 mM) upon irradiation by UV lamp at 365 nm under an O_2 atmosphere. (b) The fluorescence curves and images of TAE before and after photooxidation (λ_{ex} = 293 nm).

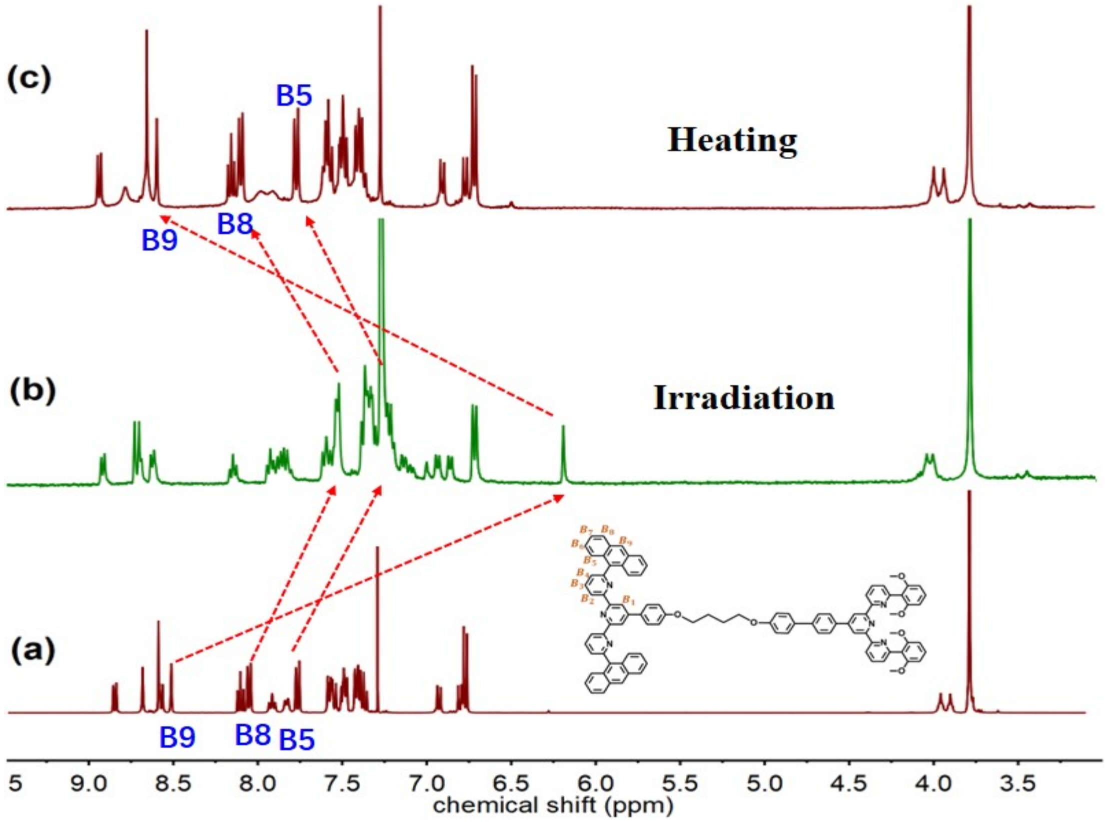

Fig. S24 ^1H NMR spectra (400 MHz, $CDCl_3$, 298 K) of the reversible photo-oxygenation of TAE ([TAE] = 5 mM) under an O_2 atmosphere: (a) before irradiation with UV light, (b) after irradiation 12 min at 365 nm, (c) after irradiation at 365 nm for 12 min and heating at 55 °C for 28 h.

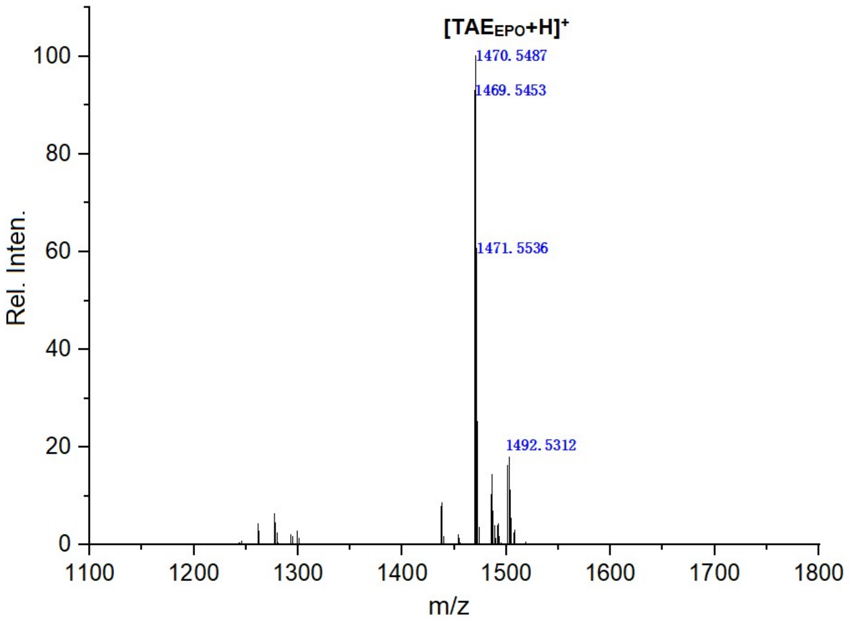

Fig. S25 High-resolution HR-ESI-MS of compound TAE_{EPO}. m/z calcd for $[TAE_{EPO}+H]^+$=1470.5416, found=1470.5487, error=4.83 ppm.

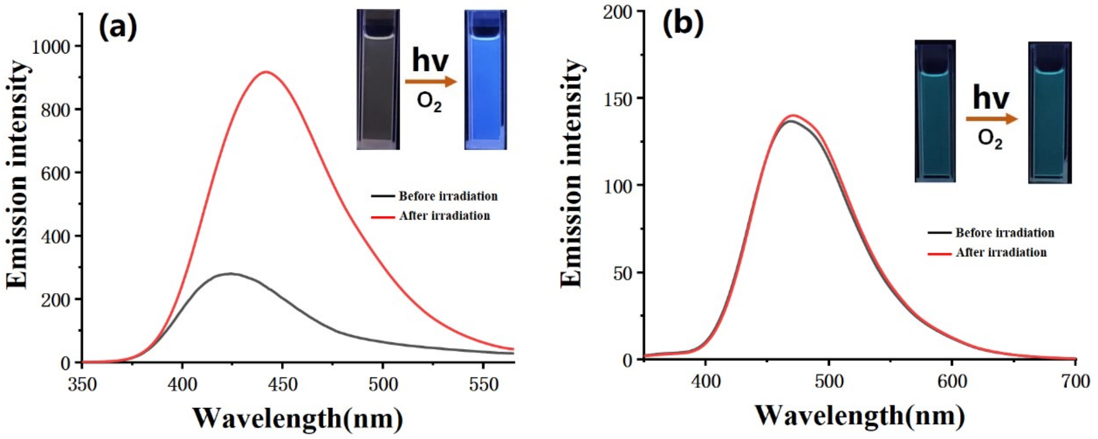

Fig. S26 The fluorescence emission spectra before UV light irradiation (black line) and after 20 min of irradiation at 365 nm (red line) for (a) 1+2+Zn^{2+}, (b) 1+3+Zn^{2+}.

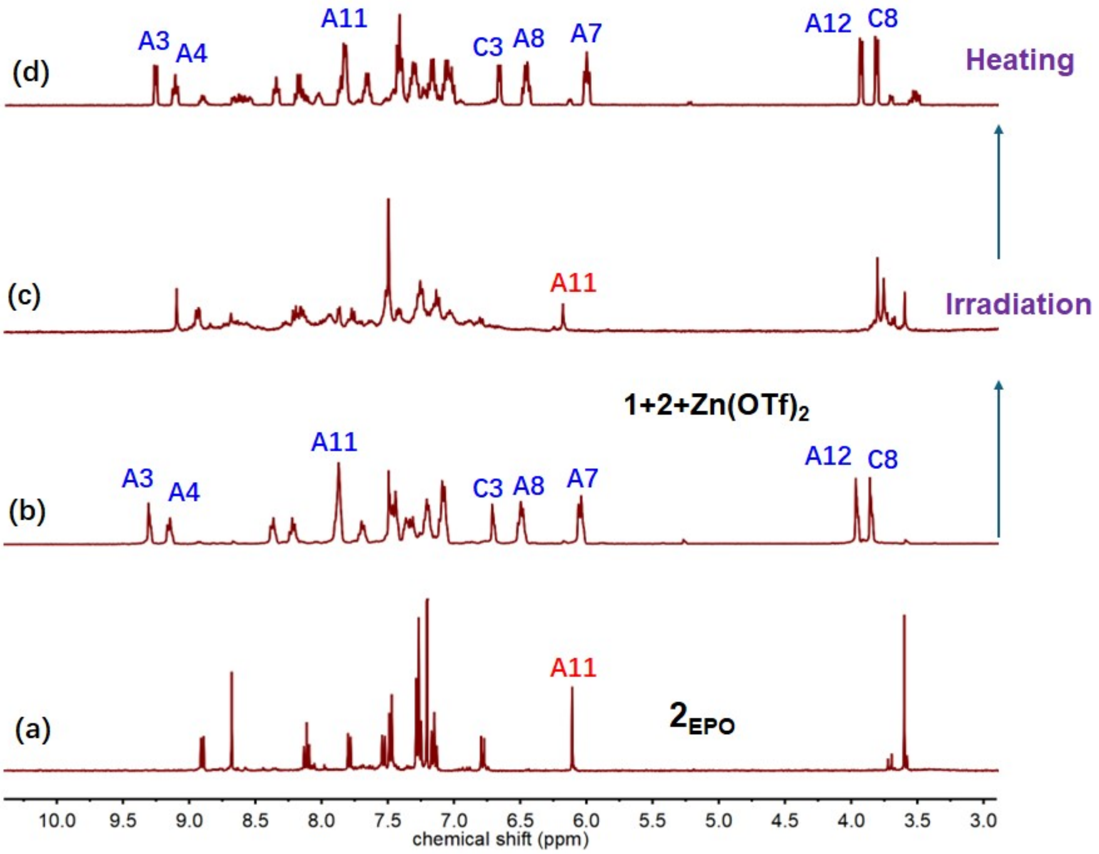

Fig. S27 ^{1}H NMR spectra (400 MHz, $CDCl_3/CD_3COCD_3$= 3/1, *v*/*v*) of (a) 2_{EPO}, (b) before irradiation with UV light under an O_2 atmosphere ([1+2+$Zn(OTf)_2$] = 5 mM), (c) after irradiation 20 min at 365 nm, (d) after irradiation at 365 nm for 20 min followed by heating at 55 °C for 28 h.

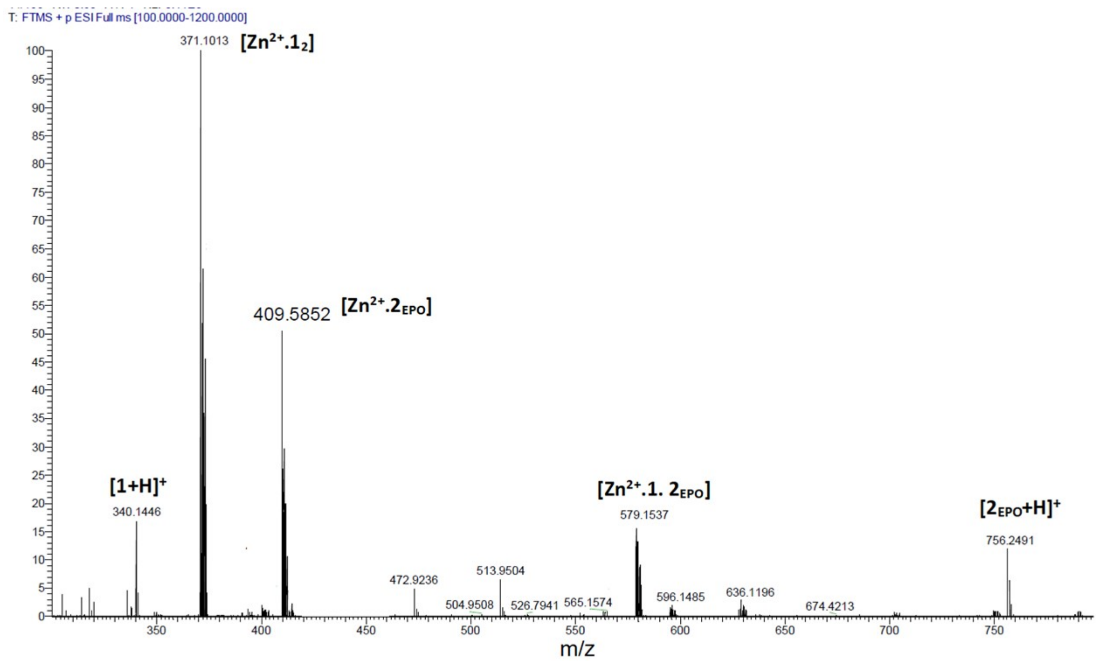

Fig. S28 HR-ESI-MS spectrum of 1+2+Zn(OTf)$_2$ after irradiation at 365 nm for 20 min in the presence of O_2.

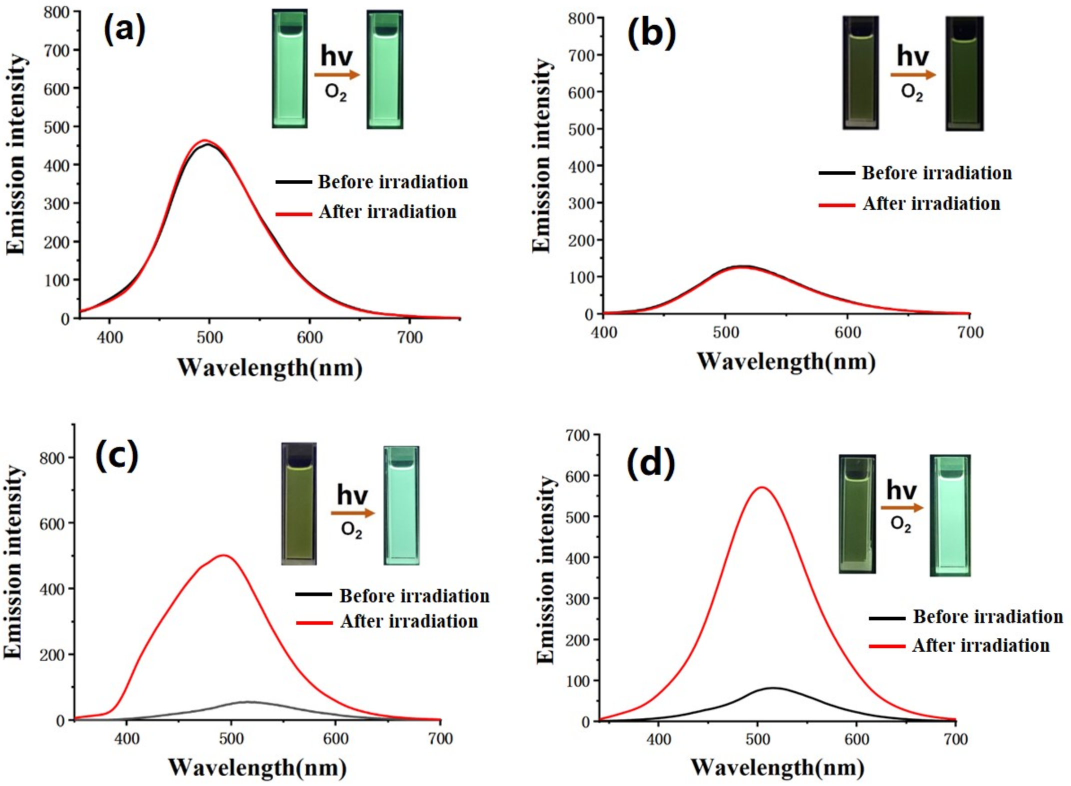

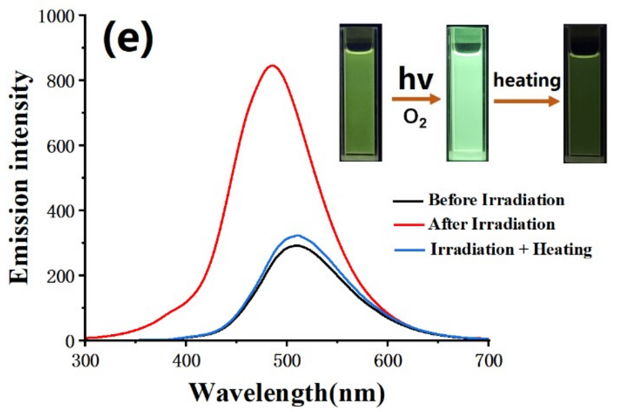

Fig. S29 Fluorescent emission spectra of (a) P5T+Zn^{2+}; (b) P5T+3+Zn^{2+}; (c) P5T+2+Zn^{2+}; (d) P5T+2+3+Zn^{2+}; (e) TAE+P5T+G2+Zn^{2+} before and after irradiation with 365 nm UV light for 20 min.

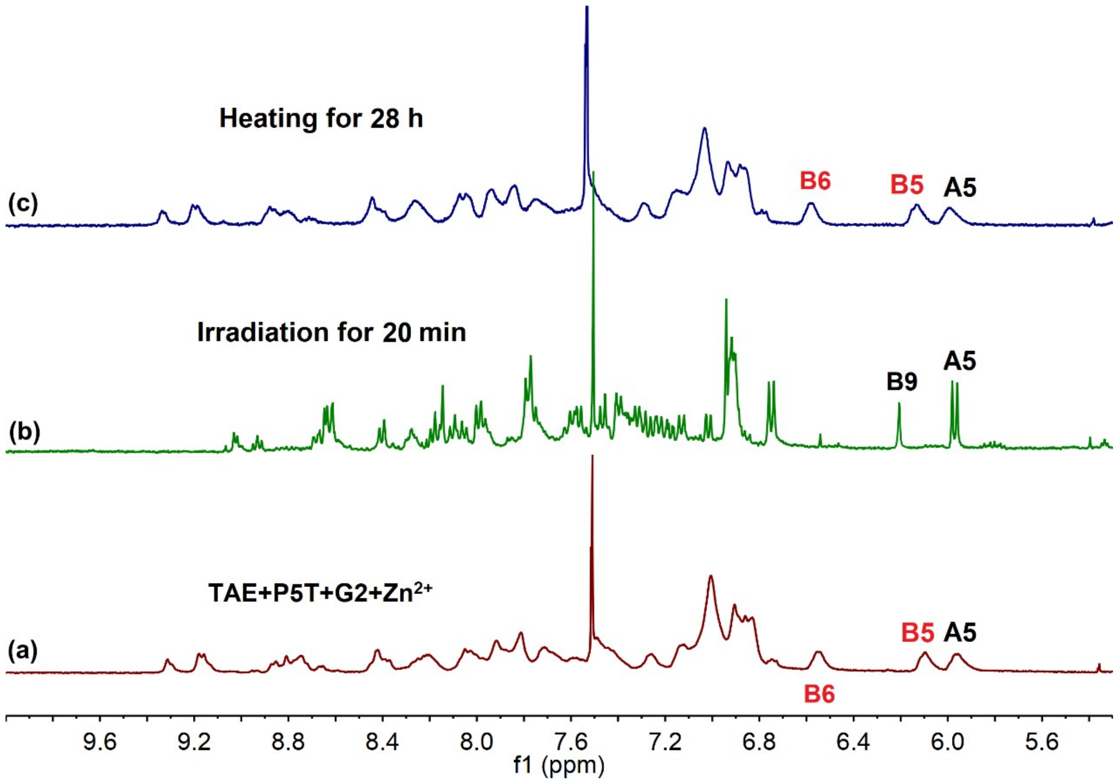

Fig. S30 ^{1}H NMR spectra (400 MHz, $CDCl_3$/CD_3COCD_3= 3/1, *v*/*v*) of the reversible photo-oxygenation of TAE+P5T+G2+$Zn(OTf)_2$ ([TAE] = 5 mM) under an O_2 atmosphere: (a) before irradiation with UV light, (b) after irradiation 20 min at 365 nm, (c) after irradiation at 365 nm for 20 min followed by heating at 55 °C for 28 h.

13. Stability investigation of host-guest complexes after UV irradiation

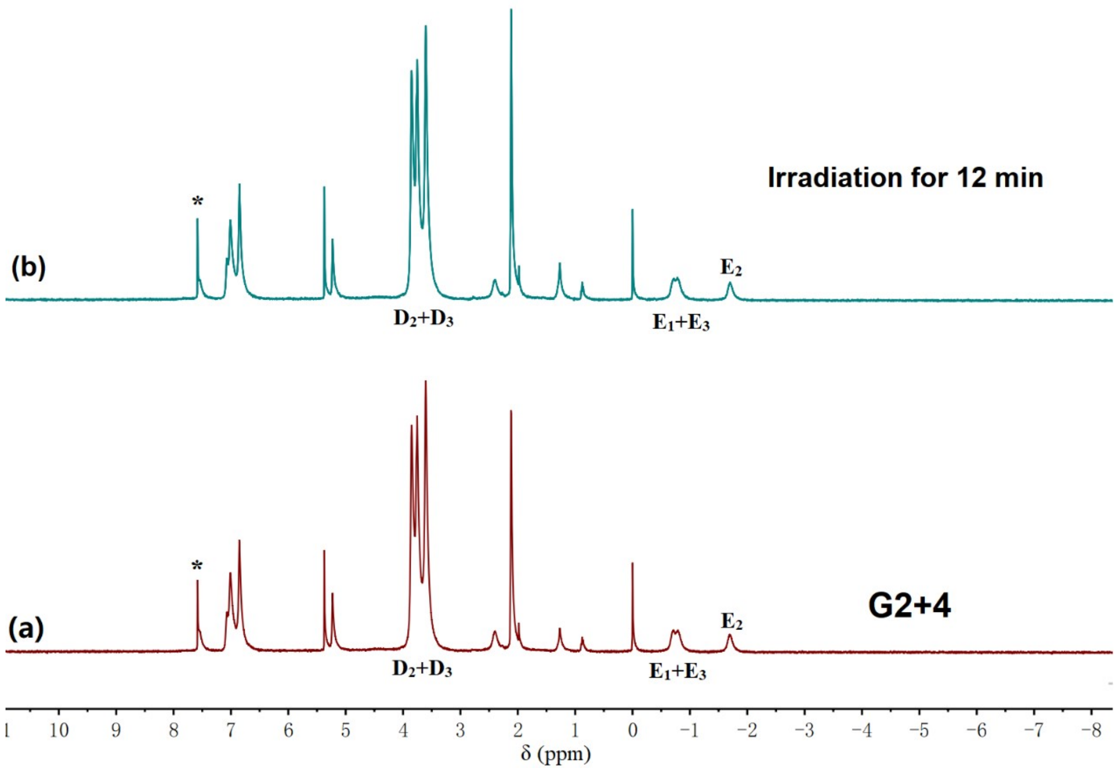

Fig. S31 ^{1}H NMR spectra (400 MHz, $CDCl_3/CD_3COCD_3$= 3:1, *v*/*v*) of G2+4: (a) before 365 nm UV irradiation ([G2+4] = 10 mM), (b) after 365 nm UV irradiation for 12 min under an O_2 atmosphere.

14. The discussion of binding constants

(1) tpy-Zn^{2+}-tay binding constant

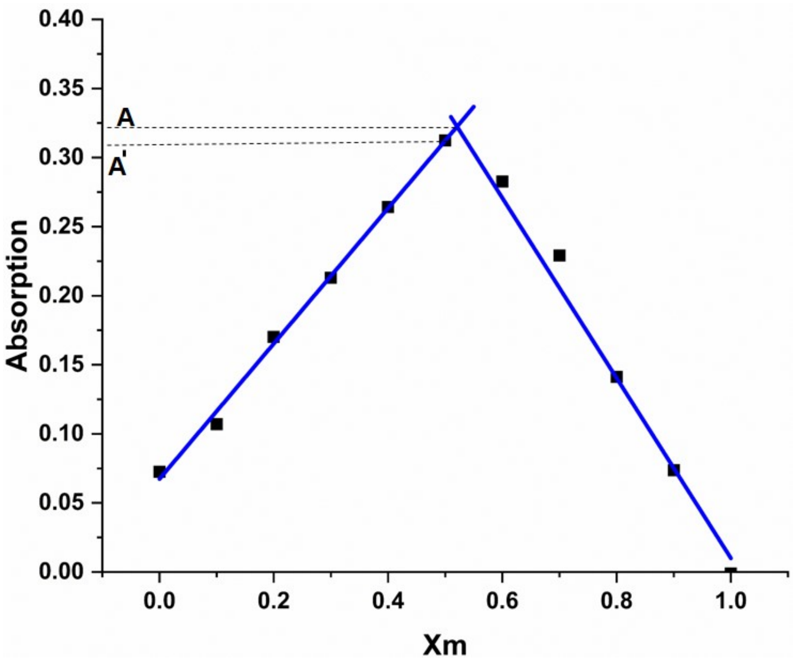

Fig. S32 Job plot of the complex formed among zinc ion, **1** (ligand) and **2** (ligand) showing a 1:1:1 stoichiometry by plotting the absorbance intensity at 339 nm against the mole fraction of zinc ion. Concentration: [**1**] = [**2**], $\frac{[1]+[2]}{2}$ + [Zn(OTf)$_2$] = 2×10^{-5}M. (chloroform/acetone =3/1, v/v, 298K).

To determine the association constant of tpy-Zn^{2+}-tay, the UV-Vis experiment (Job plot method) was conducted according to the literature method.[S2] Model compounds 1 and 2 were chosen as the ligands. A series of samples were prepared and the total molar concentration of ligands (([1]+[2])/2) and zinc ion was maintained at 2×10-5M in each sample and only the ratios of zinc ion to ligands were altered. The job plot was conducted by varying the mole fractions of the ligands (([1]+[2])/2) and zinc ion. The concentration: ([1]+[2])/2 + [Zn(OTf)$_2$] = 2×10^{-5}M. The absorbance intensity at 339 nm was plotted (Fig. S31) against the mole fraction of Zn(OTf)$_2$. The Job plot indicates a 1:1:1 binding among Zn^{2+}, 1 and 2.

Furthermore, the data of job plot were divided into two groups around X_m= 0.5. When $X_m \leq$ 0.5, the fitting equation is A= 0.4898 X_m+ 0.0674. When $X_m \geq$ 0.5, the fitting equation is A= -0.6520 X_m+ 0.6620. The intersection point of the two fitting curves is taken (X_m= 0.5208, A= 0.3239), and the experimental value is X_m= 0.5, A'= 0.3124. The dissociation degree of complex [**Zn1·2**](OTf)$_2$ was calculated from **Eq. 1**. According to the formula[S2], the dissociation degree(α) of complex [**Zn1·2**](OTf)$_2$ was calculated to be 0.036.

α= (A - A')/A, (**Eq. 1**)

The binding constant K was calculated to be 7.43×10^7 M^{-1} based on **Eq. 2**.

$$1 + Zn(OTf)_2 \rightleftharpoons [Zn\mathbf{1}](OTf)_2$$

$$\mathbf{2} + [Zn1](OTf)_2 \rightleftharpoons [Zn\mathbf{12}](OTf)_2$$

$$\boldsymbol{K} = \frac{[Zn1\cdot 2](OTf)_2}{[2]\ [Zn1](OTf)_2)} = \frac{1-\alpha}{C\alpha^2} \quad (\textbf{Eq. 2})$$

Where C is the total concentration of the complex [**Zn1·2**]$(OTf)_2$ and α is the degree of dissociation of complex [**Zn1·2**]$(OTf)_2$ when X_m value is 0.5, with the hypothesis that the ligands and zinc ion only form the complex [**Zn1·2**]$(OTf)_2$. The C is 2×10^{-5} M and the α is 0.031 when X_m is 0.5.

(2) tpy-Zn^{2+}-tey binding constant

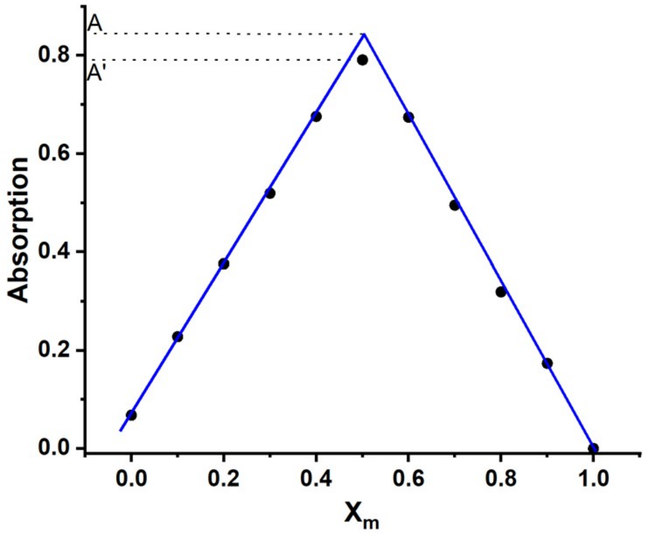

Fig. S33 Job plot of the complex formed among zinc ion, **1** (ligand) and **3** (ligand) showing a 1:1:1 stoichiometry by plotting the absorbance intensity at 348 nm against the mole fraction of zinc ion. Concentration: [**1**] = [**3**], $\frac{[1]+[3]}{2}$ + [Zn(OTf)$_2$] = 2×10^{-5}M. (chloroform/acetone =3/1, v/v, 298K).

To determine the association constant of tpy-Zn^{2+}-tey, the UV-Vis experiment (Job plot method) was conducted according to the literature method.[S2] Model compounds 1 and 3 were chosen as the ligands. A series of samples were prepared and the total molar concentration of ligands (([1]+[3])/2) and zinc ion was maintained at 2×10^{-5} M in each sample and only the ratios of zinc ion to ligands were altered. The job plot was conducted by varying the mole fractions of the ligands (([1]+[2])/2) and zinc ion. The concentration: ([1]+[3])/2 + [Zn(OTf)$_2$] = 2×10^{-5}M. The absorbance intensity at 348 nm was plotted (Fig. S32) against the mole fraction of Zn(OTf)$_2$. The Job plot indicates a 1:1:1 binding among Zn^{2+}, 1 and 3.

Furthermore, the data of job plot were divided into two groups around X_m= 0.5. When $X_m \leq$ 0.5, the fitting equation is A= 1.458X_m+ 0.0785. When $X_m \geq$ 0.5, the fitting equation is A= -1.609 X_m+ 1.6161. The intersection point of the two fitting curves is taken (X_m= 0.5235, A= 0.836), and the experimental value is X_m= 0.5, A'= 0.791. The degree of dissociation of complex [**Zn1·3**]$(OTf)_2$ was calculated from **Eq. 3**. According to the formula[S2], the dissociation degree(α) of complex [**Zn1·3**]$(OTf)_2$ was calculated to be 0.0538.

α= (A - A')/A, (**Eq. 1**)

The binding constant K was calculated to be 3.27×10^{7} M^{-1} based on **Eq. 3**.

$$\mathbf{1} + Zn(OTf)_2 \rightleftharpoons [Zn\mathbf{1}](OTf)_2$$

$$\mathbf{3} + [Zn1](OTf)_2 \rightleftharpoons [Zn\mathbf{13}](OTf)_2$$

$$\boldsymbol{K} = \frac{[Zn1\cdot3](OTf)_2}{[3]\,[Zn1](OTf)_2)} = \frac{1-\alpha}{C\alpha^2} \quad (\textbf{Eq. 3})$$

Where C is the total concentration of the complex [Zn**1·3**]$(OTf)_2$ and α is the degree of dissociation of complex [**Zn1·3**]$(OTf)_2$ when X_m value is 0.5, with the hypothesis that the ligands and zinc ion only form the complex [**Zn1·3**]$(OTf)_2$. The C is 1×10^{-5} M and the α is 0.0538 when X_m is 0.5.

(3) tpy-Zn^{2+}-tpy binding constant

The binding constant of tpy-Zn^{2+}-tpy was referred to the reported literature, The Ka is about 7.76×10^{6} M^{-1}.[S3]

(4) P5-TPN binding constant

The binding constant of P5-TPN was referred to the reported literature, The Ka is about 9.03×10^{3} M^{-1}.[S3]

15. Synthesis of intermediates and monomers

(1) The synthesis of monomer TAE

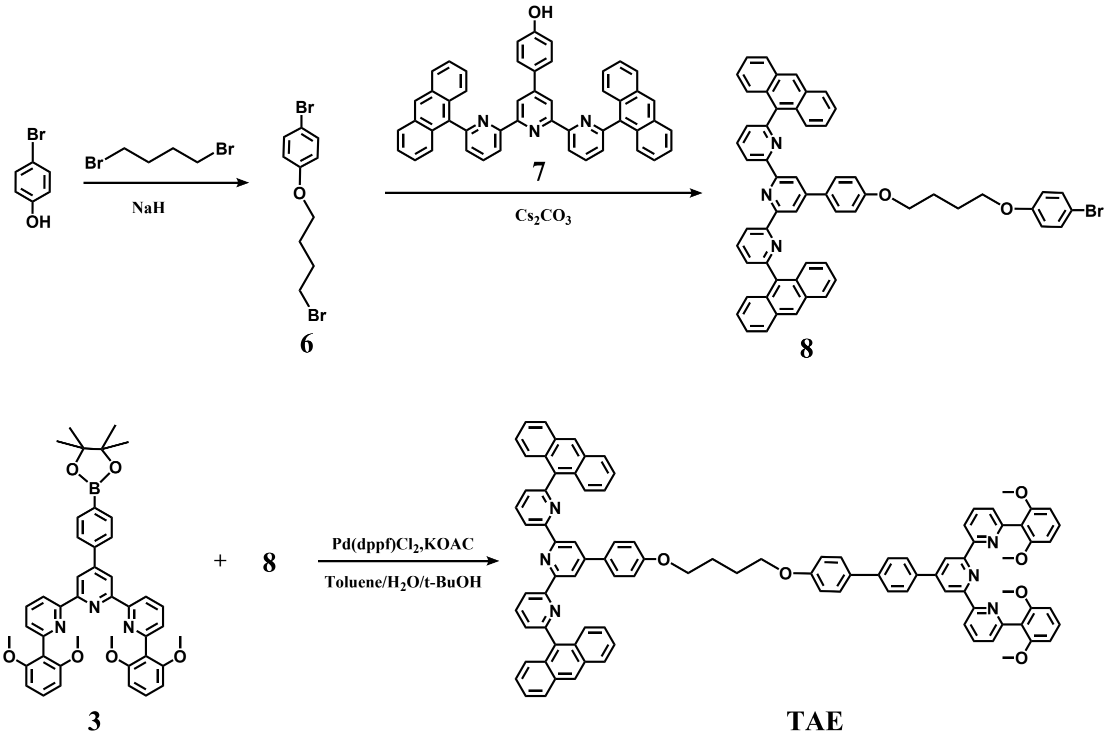

Scheme S1 Synthetic route of the heteroditopic monomer TAE

Compound **3** was synthesized according to the literature procedure[S4], and Compound 7 was synthesized according to the literature procedure[S5].

Synthesis of Compound 6

4-bromophenol (5.00 g, 28.90 mmol), 1,4-dibromobutane (31.20 g, 144.50 mmol), sodium hydride (3.60 g, 86.70 mmol, 60%wt), and DMF (120ml) were added into a 250 ml round–bottom flask, the mixture was stirred at 80°C for 10 h. The reaction mixture was cooled to room temperature and the solvent was evaporated under reduced pressure. The crude product was purified by flash column chromatography (PE/EtOH =200/1, v/v) to give compound **6** (7.40 g, 83 %) as a colorless oily liquid.

^{1}H NMR (400 MHz, $CDCl_3$) δ (ppm)= 7.42-7.31 (m, 2H), 6.81-6.72 (m, 2H), 3.95 (t, J = 6.1 Hz, 2H), 3.48 (t, J = 6.5 Hz, 2H), 2.00 (s, 2H), 1.93 (t, J = 7.2Hz, 2H).

^{13}C NMR (100 MHz, $CDCl_3$) δ (ppm)= 160.36, 132.95, 116.80, 111.80, 64.87, 33.46, 30.71, 27.26.

HR-ESI-MS: m/z calcd for $[M+H]^+$=691.16039, found=691.15859, error=2.0 ppm.

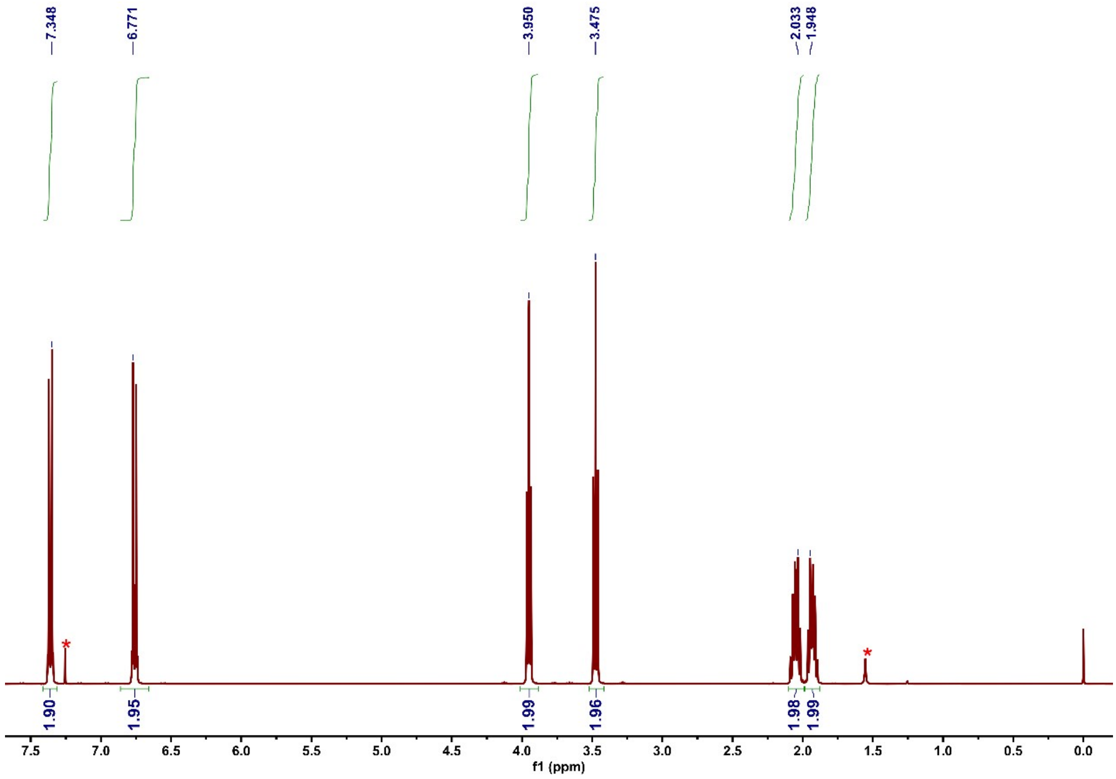

Fig S34 ^{1}H NMR spectrum (400 MHz, $CDCl_3$, 298 K) of compound 6.

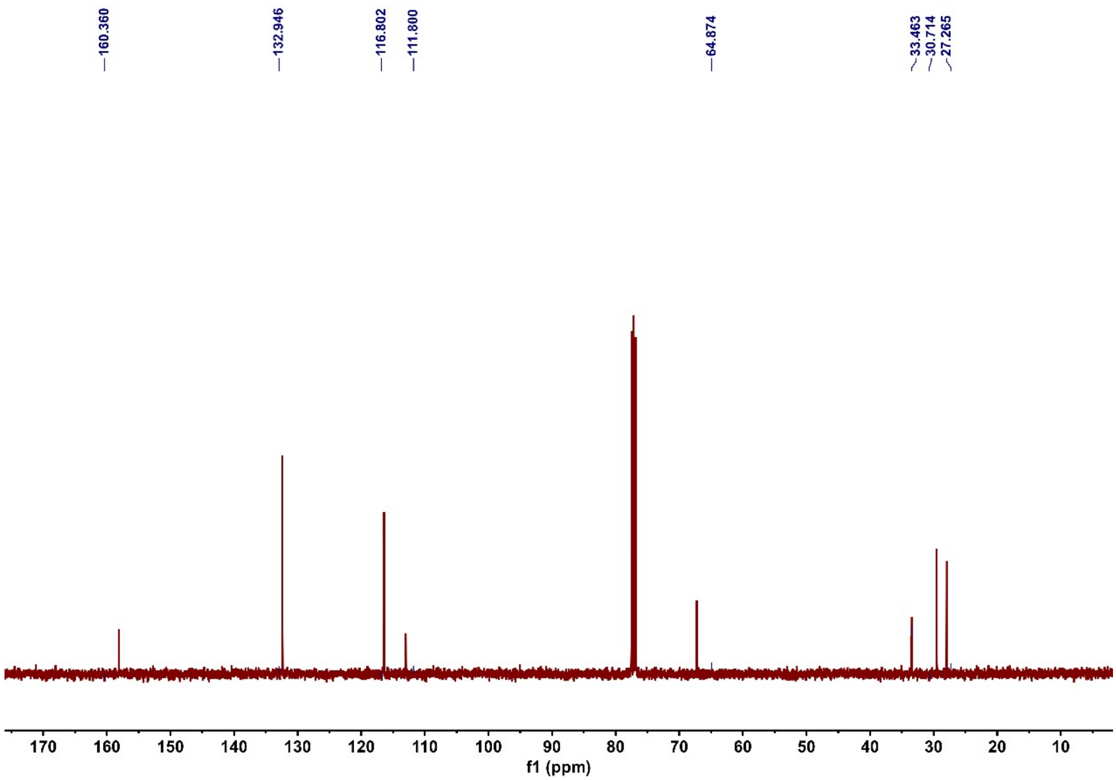

Fig S35 ^{13}C NMR spectrum (100 MHz, $CDCl_3$, 298 K) of compound 6.

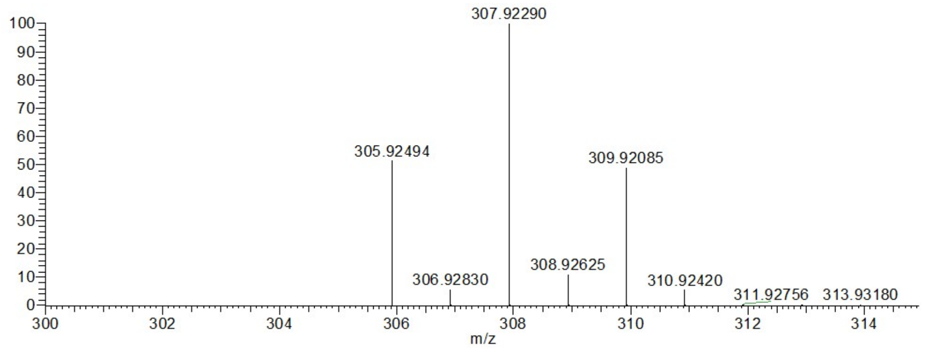

Fig S36 High-resolution HR-ESI-MS mass spectrum of compound 6.

Synthesis of Compound 8

Compound **6** (0.68 g, 2.21 mmol), compound **7** (1.00 g,1.48 mmol), cesium carbonate (1.44 g,4.43 mmol), and DMF (120 mL) were added into a 250 mL round–bottom flask, the mixture was stirred at 80°C for 11 h. The reaction mixture was cooled to room temperature and the solvent was evaporated under reduced pressure. The crude product was purified by flash column chromatography (PE/CH_2Cl_2 =1/2, v/v) to give compound 8 (0.96 g, 72%) as a white solid.

^{1}H NMR (400 MHz, $CDCl_3$) δ (ppm)= 8.97 (d, *J* = 7.7 Hz, 2H), 8.70 (s, 2H), 8.60 (s, 2H), 8.17 (t, *J* = 7.7 Hz, 2H), 8.11 (d, *J* = 8.4 Hz, 4H), 7.79 (d, *J* = 8.7 Hz, 4H), 7.59 (t, *J* = 7.9 Hz, 4H), 7.50 (d, *J* = 8.2 Hz, 4H), 7.41 (d, *J* = 8.0Hz, 4H), 7.36-7.30 (m, 2H), 6.81-6.66 (m, 4H), 3.89 (d, *J* = 5.9, 4H), 1.91-1.83 (m, 4H).

^{13}C NMR (100 MHz, $CDCl_3$) δ (ppm) = 159.65, 158.03, 157.60, 156.63, 155.85, 137.12, 135.55, 132.18, 131.47, 130.20, 128.54, 128.48, 127.53, 127.00, 126.36, 125.82, 125.20, 120.08, 118.96, 116.26, 114.63, 67.62, 25.79.

HR-ESI-MS: m/z calcd for $[M+H]^+$=904.25332, found=904.25331, error=0.1 ppm.

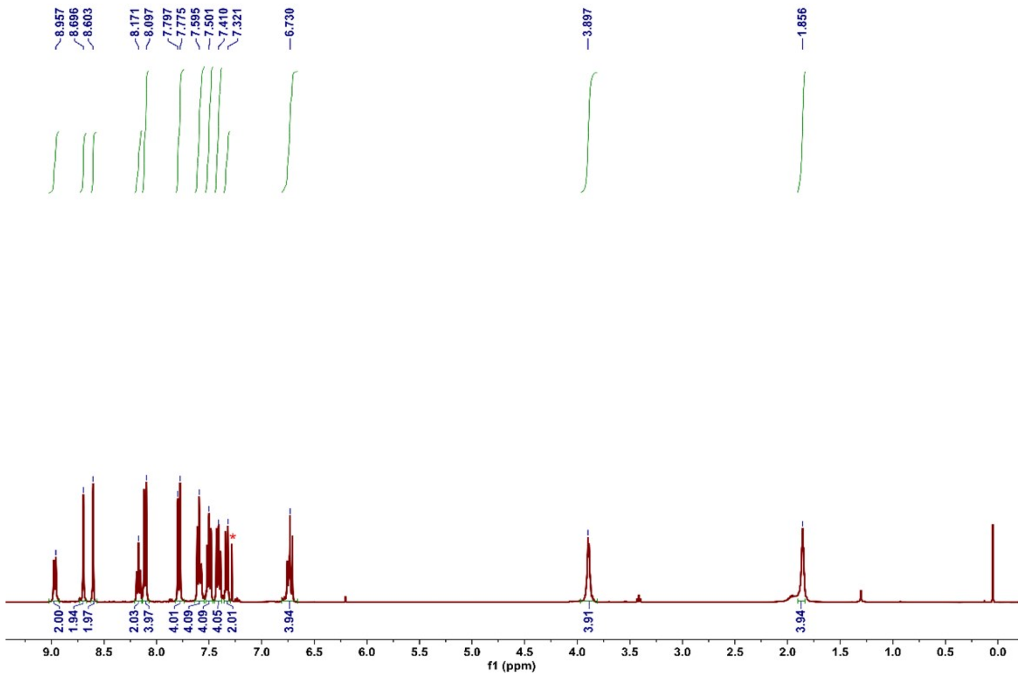

Fig S37 ^{1}H NMR spectrum (400 MHz, $CDCl_3$, 298 K) of compound 8.

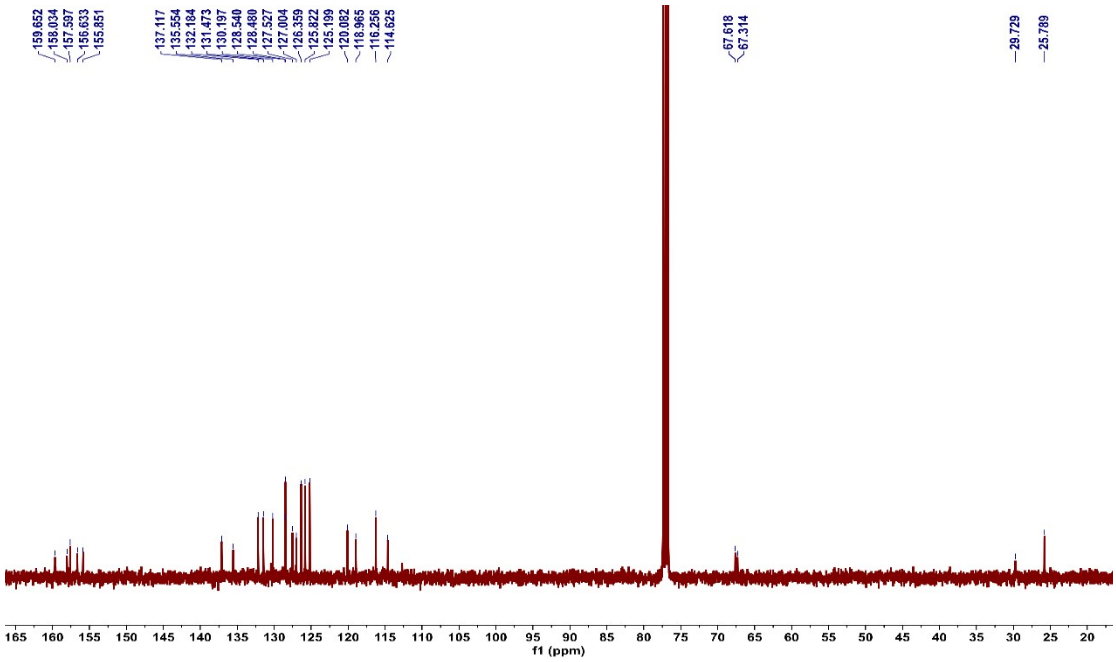

Fig S38 ^{13}C NMR spectrum (400 MHz, $CDCl_3$, 298 K) of compound 8.

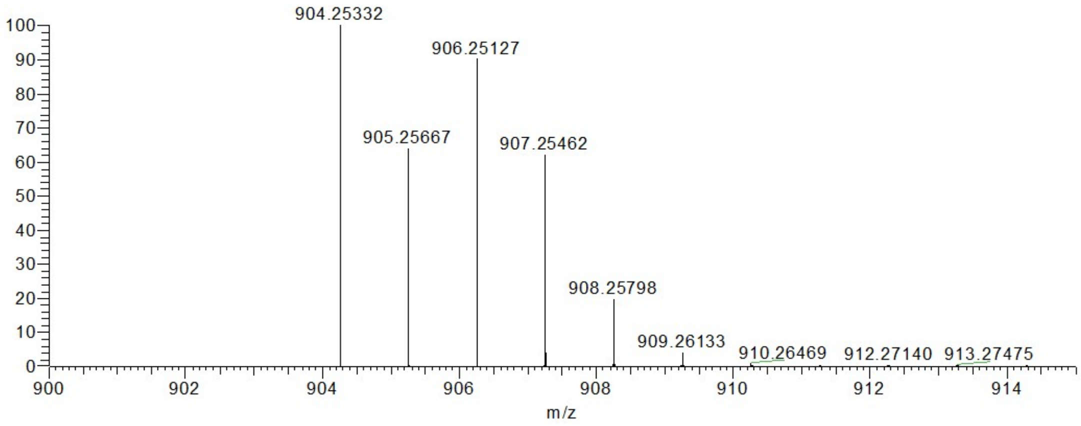

Fig S39 High-resolution HR-ESI-MS mass spectrum of compound 8.

Synthesis of Compound TAE

Compound **8** (0.70 g, 0.77 mmol), compound **3** (0.74 g, 1.04 mmol), sodium carbonate (0.49 g, 4.64 mmol), [1,1'-bis(diphenylphosphino)ferrocene]dichloropalladium(II) (0.12 g, 0.017 mmol), and a mixed solvent of toluene/H_2O/t-BuOH (3:3:1, v/v, 63 mL) were added into a 100 mL round–bottom flask in nitrogen atmosphere, the reaction mixture was then refluxed at 80℃ for 16 h under N_2. After the reaction mixture was cooled to room temperature. the reaction mixture was dissolved in CH_2Cl_2 (150 mL) and washed 3 times with water (50 mL each time). The organic phases were combined and dried over anhydrous Na_2SO_4, the solvent was evaporated to afford

the crude product, the crude product was purified by flash column chromatography (PE/EA =1/4, v/v, and CH_2Cl_2) to give compound TAE (0.85 g,78%) as a yellow solid.

^{1}H NMR (400 MHz, $CDCl_3$) δ (ppm) = 8.91 (d, *J* = 7.8, 1.0 Hz, 2H), 8.73 (s, 2H), 8.65-8.59 (m, 4H), 8.55 (s, 2H), 8.11 (t, *J* = 7.8 Hz, 2H), 8.05 (d, *J* = 8.4 Hz, 4H), 7.91 (t, *J* = 7.8 Hz, 2H), 7.82 (d, *J* = 8.0 Hz, 2H), 7.75 (d, *J* = 8.8 Hz, 4H), 7.57 – 7.49 (m, 6H), 7.48 – 7.41 (m, 6H), 7.39 – 7.30 (m, 8H), 6.88 – 6.82 (m, 2H), 6.69 (dd, *J* = 8.8, 8.6 Hz, 6H), 3.96 – 3.84 (m, 4H), 3.74 (s, 12H), 1.84 (t, *J* = 3.3 Hz, 4H).

^{13}C NMR (100 MHz, $CDCl_3$) δ (ppm) =159.66, 157.60, 156.83, 137.12, 135.66, 132.74, 131.50, 130.23, 129.70, 128.53, 128.44, 127.97, 127.67, 126.96, 126.85, 126.39, 125.87, 125.24, 120.04, 119.60, 119.10, 118.87, 114.78, 114.64, 104.68, 68.03, 67.41, 56.20, 25.82, 25.67.

HR-ESI-MS: m/z calcd for $[M+H]^+$=1406.5620, found=1406.5630, error=0.1 ppm.

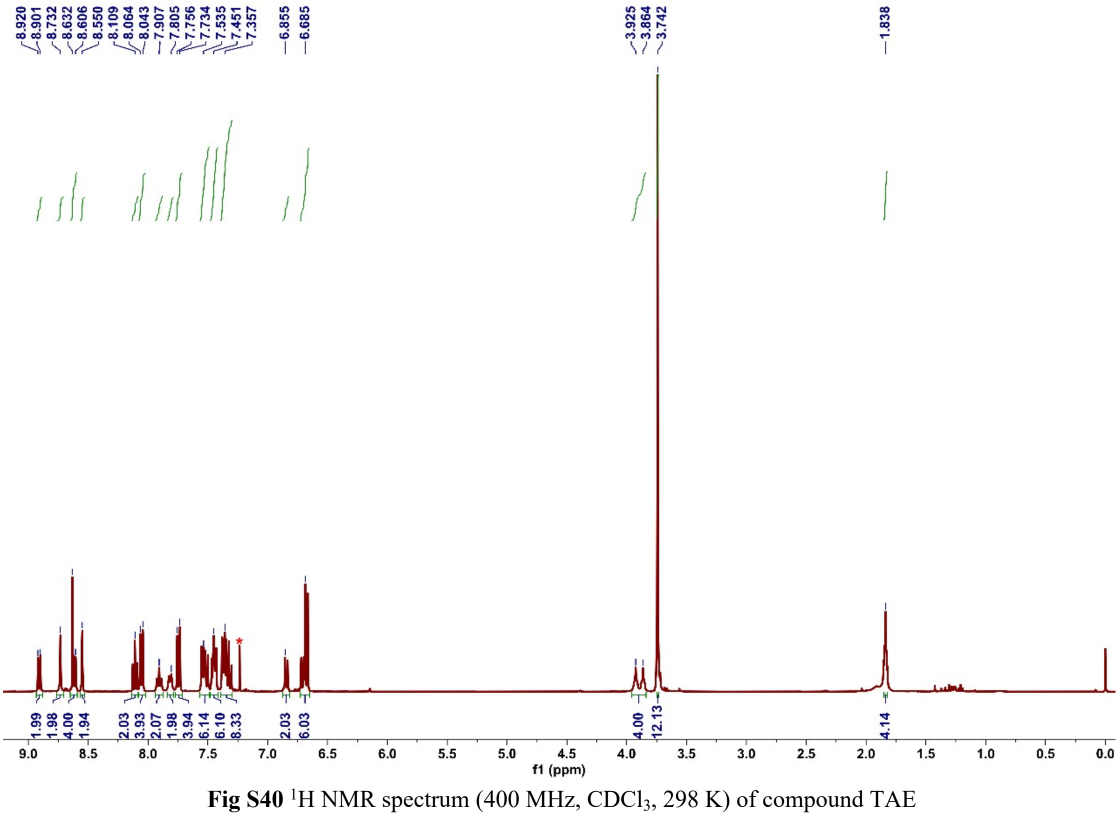

Fig S40 ^{1}H NMR spectrum (400 MHz, $CDCl_3$, 298 K) of compound TAE

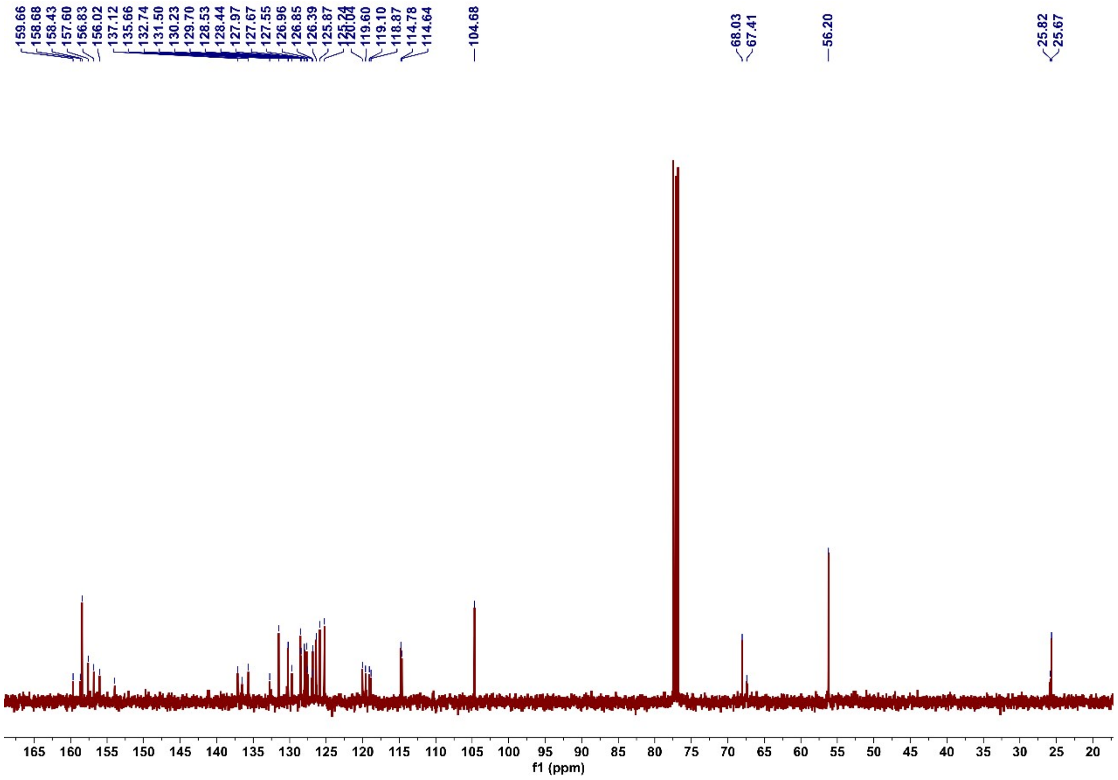

Fig S41 ^{13}C NMR spectrum (400 MHz, $CDCl_3$, 298 K) of compound TAE

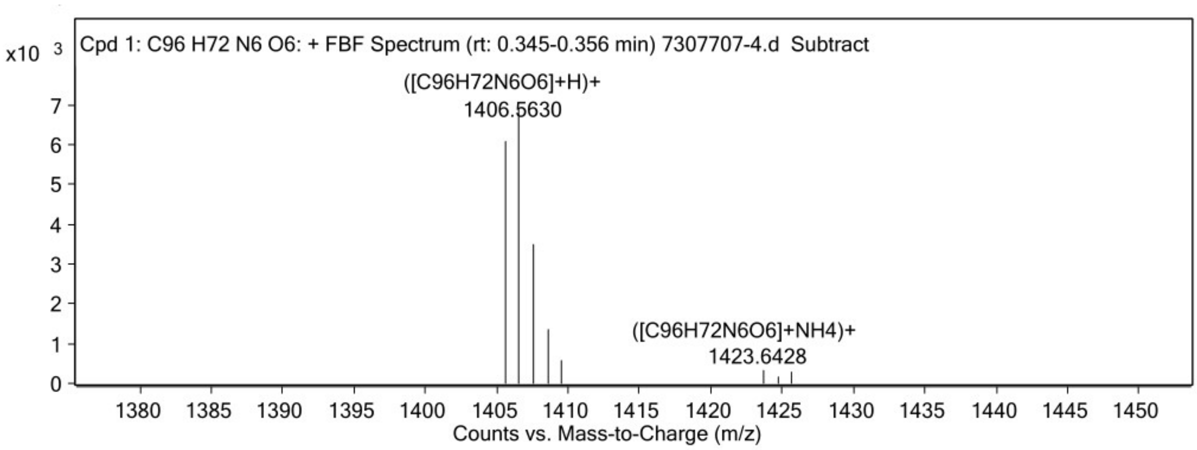

Fig S42 High-resolution HR-ESI-MS mass spectrum of compound TAE

(2) The synthesis of monomer P5T

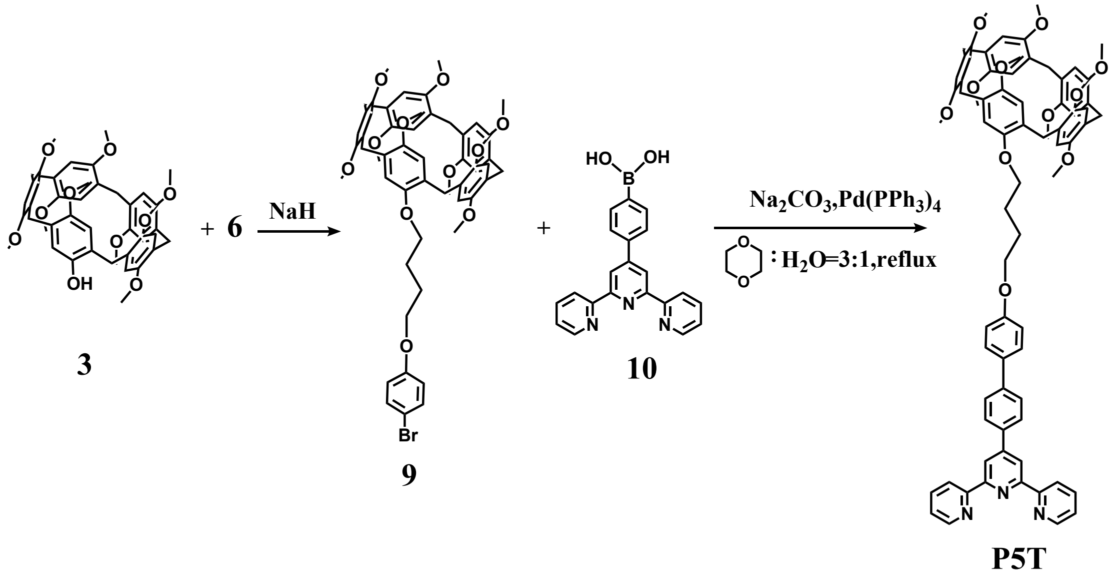

Scheme S2 Synthetic route of the heteroditopic monomer P5T

Compound **3** was synthesized according to the literature procedure,[S6] and compound 10 was synthesized according to the literature procedure.[S7]

Synthesis of Compound 9

Compound **6** (3.00 g, 4.07 mmol), compound **3** (1.50 g, 4.89mmol), sodium hydride (0.29 g, 12.21 mmol, 60%wt), DMF (120mL) were added into a 100 mL round–bottom flask under a nitrogen atmosphere. After the reaction mixture was stirred at 80°C for 11 h, the reaction mixture was cooled to room temperature and the solvent was evaporated under reduced pressure. The crude product was purified by flash column chromatography (PE/ CH_2Cl_2 =1/1, v/v) to give compound **9** (2.90 g, 74%) as a white solid.

^{1}H NMR (400 MHz, $CDCl_3$) δ (ppm) =7.38-7.33 (m, 2H), 6.84-6.68 (m, 12H), 4.95 (s, 1H), 3.91 (dt, J = 18.8, 5.6 Hz, 4H), 3.83-3.56 (m, 36H), 2.02-1.80 (m, 4H).

^{13}C NMR (101 MHz, $CDCl_3$) δ (ppm) =158.13, 150.83, 150.80, 149.94, 132.24, 128.33, 128.28, 116.27, 114.96, 114.15, 112.73, 67.94, 67.76, 55.87, 55.82, 55.79, 55.77, 55.74, 52.94, 29.70, 29.63, 29.55, 26.34, 26.14.

HR-ESI-MS: m/z calcd for $[M+H]^+$=963.3314, found=963.3317, error=0.1 ppm.

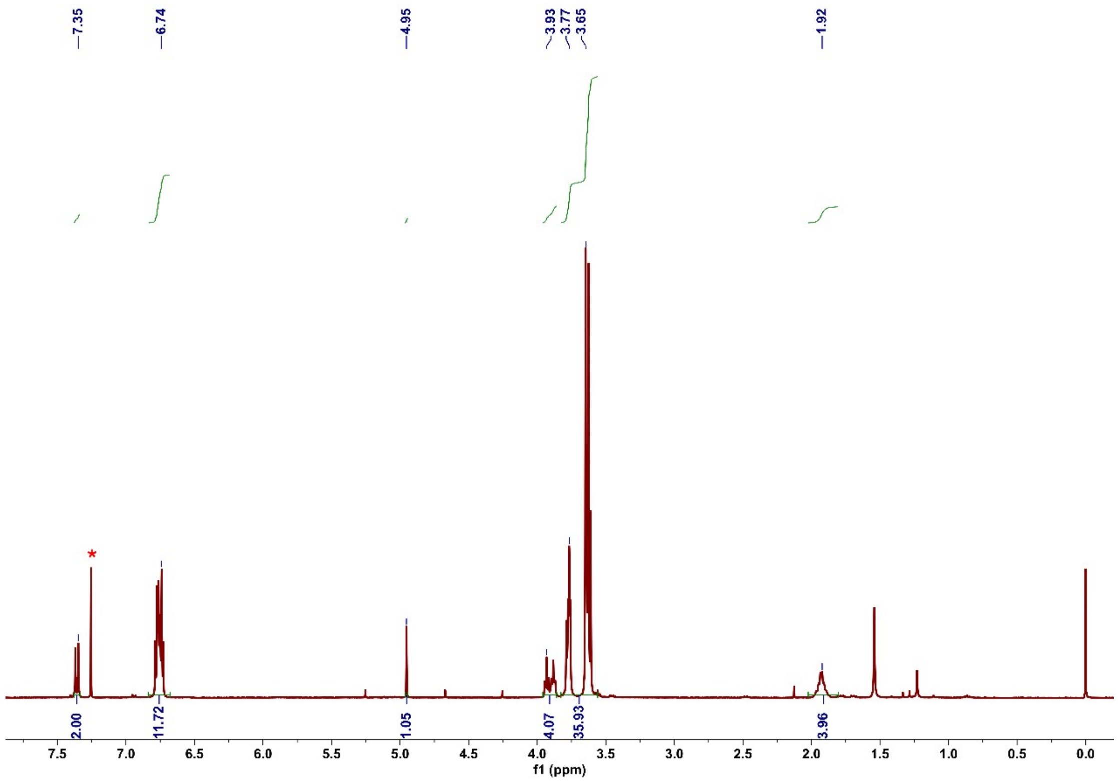

Fig S43 ^{1}H NMR spectrum (400 MHz, $CDCl_3$, 298 K) of compound 9

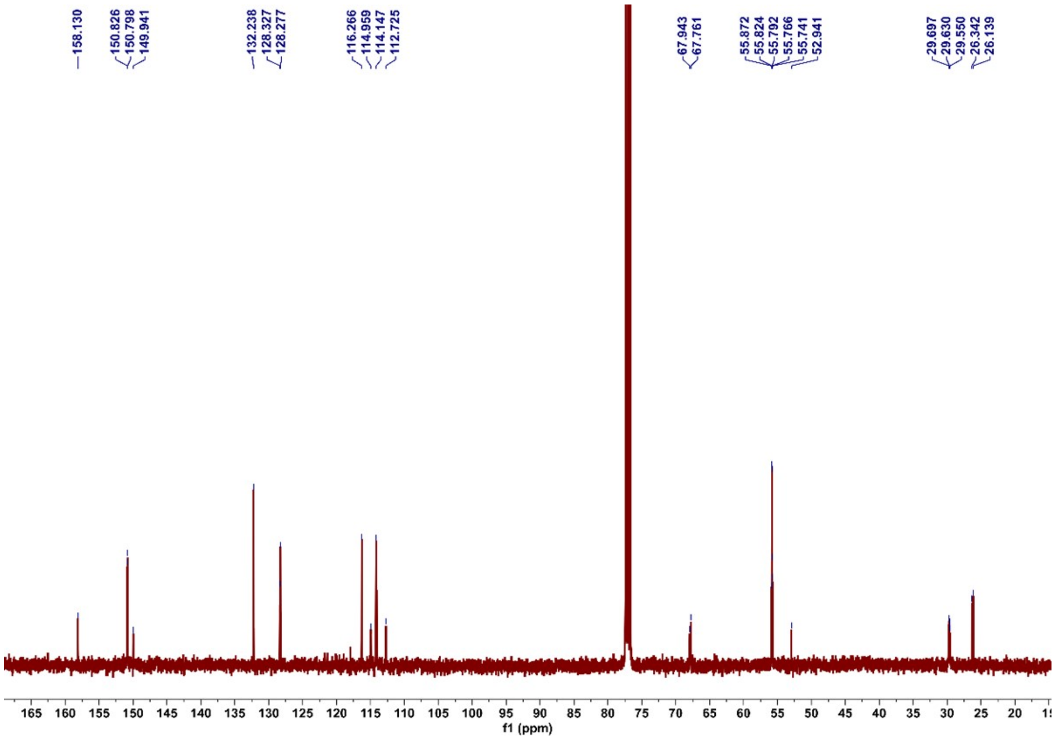

Fig S44 ^{13}C NMR spectrum (400 MHz, $CDCl_3$, 298 K) of compound 9

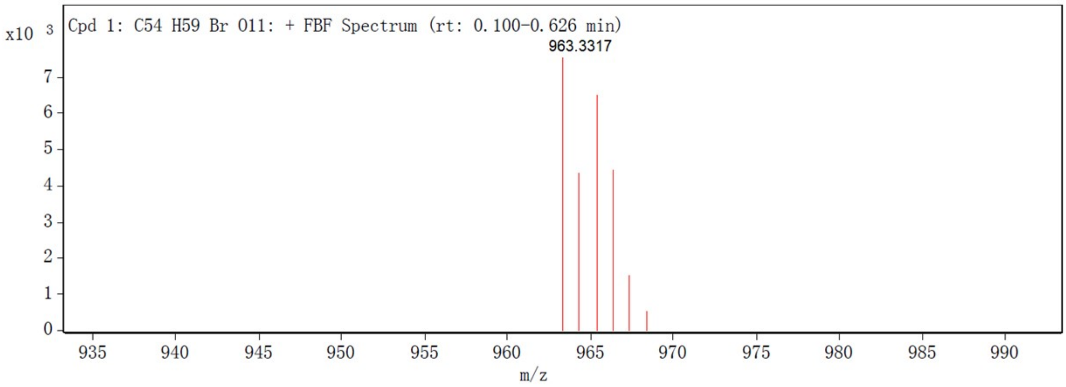

Fig S45 High-resolution HR-ESI-MS mass spectrum of compound 9

Synthesis of Compound P5T

Compound **9** (2.00 g, 2.07 mmol), compound **10** (1.10 g, 6.22 mmol), sodium carbonate (0.66 g, 6.22 mmol), tetrakis(triphenylphosphine) palladium (0.24 g, 0.21 mmol), and a mixed solvent of 1,4-dioxane /H_2O (11:1, v/v, 120 ml) were added into a 250 mL round–bottom flask under N2 atmosphere. The mixture was then refluxed at 80℃ for 15 h under N_2 atmosphere. The reaction mixture was cooled to room temperature and the solvent was evaporated under reduced pressure. The crude product was purified by flash column chromatography (PE/EA=1/4, v/v) to give compound **P5T** (1.85 g, 75%) as a white solid.

^{1}H NMR (400 MHz, $CDCl_3$) δ (ppm) = 8.85 (s, 2H), 8.79 (dt, *J* = 4.7, 1.6 Hz, 2H), 8.74 (dt, *J* = 8.0, 1.1 Hz, 2H), 8.08 – 8.00 (m, 2H), 7.94 (td, *J* = 7.7, 1.8 Hz, 2H), 7.78 – 7.70 (m, 2H), 7.68 – 7.60 (m, 2H), 7.41 (ddd, *J* = 7.4, 4.8, 1.2 Hz, 2H), 7.07 – 6.99 (m, 2H), 6.80 (dd, *J* = 10.9, 4.8 Hz, 10H), 4.11 (t, *J* = 5.9 Hz, 2H), 3.95 (t, *J* = 5.7 Hz, 2H), 3.89 – 3.76 (m, 10H), 3.68 (d, *J* = 4.6 Hz, 26H), 2.12 – 1.94 (m, 4H).

^{13}C NMR (100 MHz, $CDCl_3$) δ(ppm) = 158.91, 156.23, 155.89, 150.83, 149.86, 149.09, 137.03, 128.26, 128.19, 127.74, 127.12, 123.90, 121.47, 118.71, 114.87, 114.16, 67.67, 55.82, 55.78, 29.77, 26.50, 26.34.

HR-ESI-MS: m/z calcd for $[M+H]^+$=1192.5318, found=1192.5291, error=2.26 ppm.

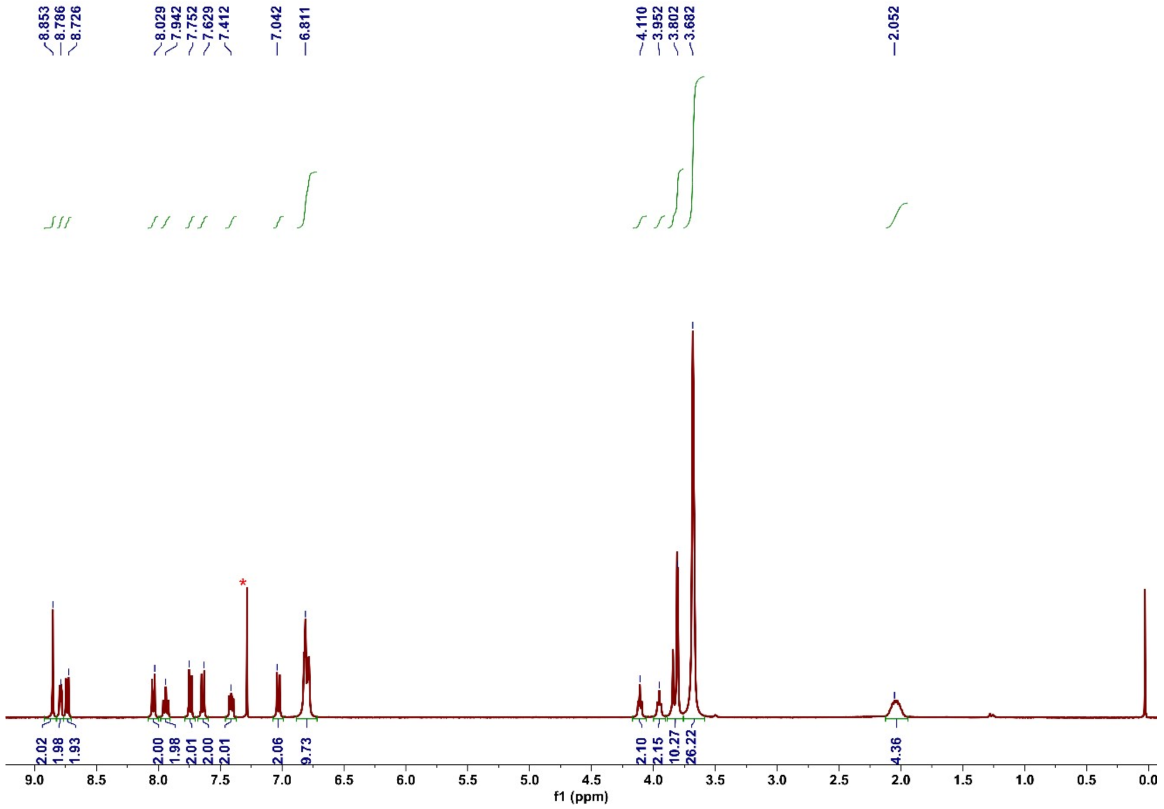

Fig S46 ^{1}H NMR spectrum (400 MHz, $CDCl_3$, 298 K) of compound P5T

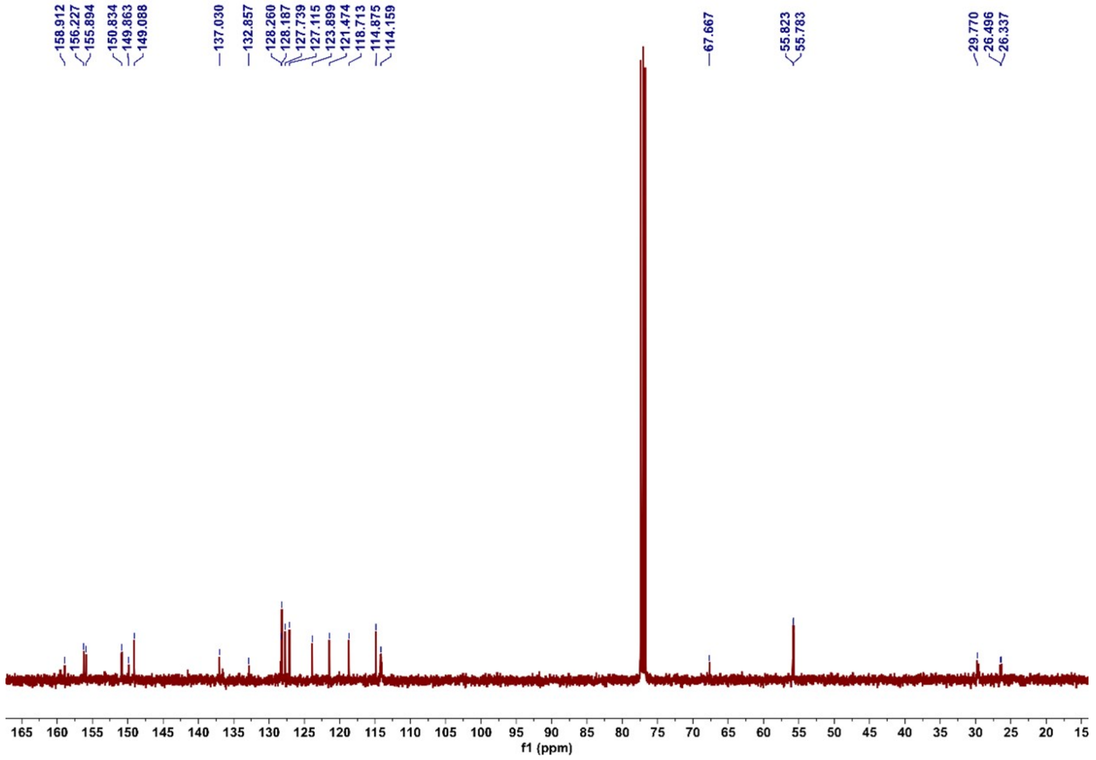

Fig. S47 ^{13}C NMR spectrum (400 MHz, $CDCl_3$, 298 K) of compound P5T

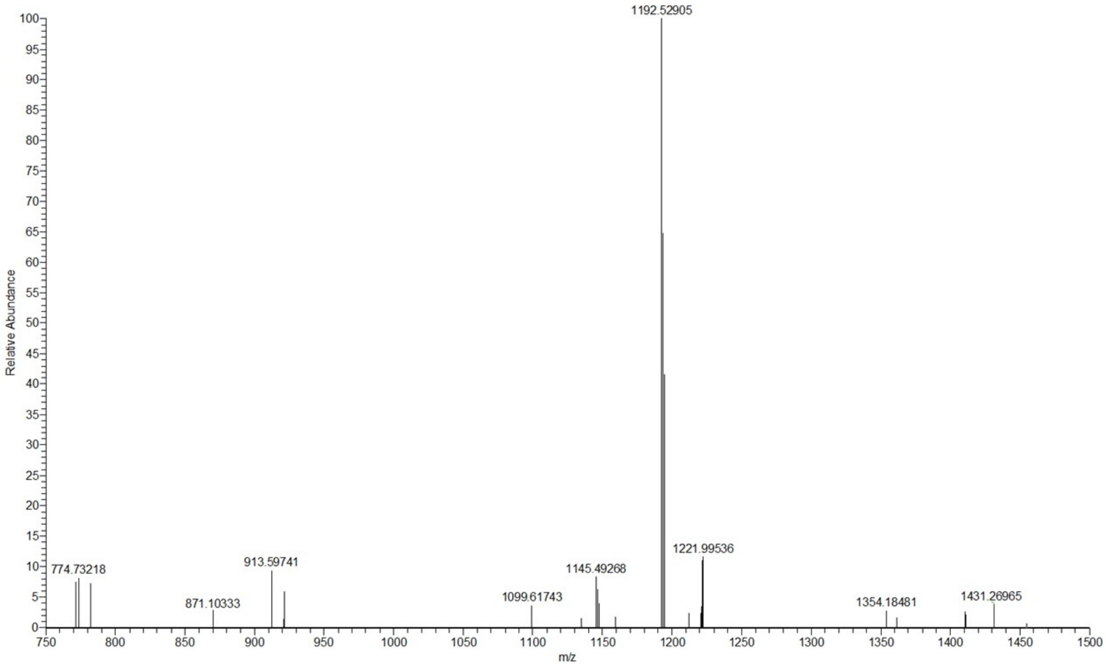

Fig. S48 High-resolution HR-ESI-MS mass spectrum of compound P5T

(3) The synthesis of monomer G2

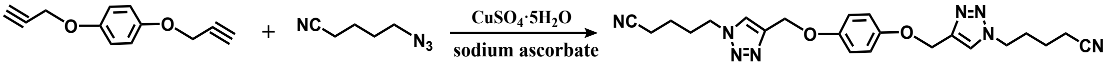

10 **11** **G2**

Scheme S3 Synthetic route of the heteroditopic monomer G2

compound 10 was synthesized according to the literature procedure[S8]

Synthesis of Compound G2

Compound 10 (1.40 g, 7.52 mmol), compound 11 (2.33 g, 18.80 mmol), copper(II) sulfate pentahydrate (0.94 g, 3.76 mmol), sodium ascorbate (2.20 g, 11.10 mmol), and a mixed solvent system (60 mL) of THF/H_2O (5:1, v/v) were charged into a 100 mL round-bottom flask. The reaction mixture was stirred at 70 °C for 12 h under an N_2 atmosphere. After cooling to ambient temperature, the mixture was dissolved in CH_2Cl_2 (100 mL) and extracted twice with H_2O (100 mL). The combined organic layers were dried over anhydrous Na_2SO_4, and the solvent was removed under reduced pressure to yield the crude product. Purification by flash column chromatography (CH_2Cl_2/CH_3CN = 3/1, v/v) followed by concentration of the product-containing fractions under vacuum afforded compound G2 as a white solid (2.52 g, 77%).

^{1}H NMR (400 MHz, $CDCl_3$) δ (ppm) = 7.63 (s, 2H), 6.94 (s, 4H), 5.19 (s, 4H), 4.45 (t, J = 6.8 Hz, 4H), 2.42 (t, J = 7.0 Hz, 4H), 2.12 (m, 4H), 1.74-1.69 (m, 4H).

^{13}C NMR (100 MHz, $CDCl_3$) δ (ppm) = 152.79, 144.52, 122.77, 119.00, 115.93, 62.61, 49.26, 29.04, 22.34, 16.67.

HR-ESI-MS: m/z calcd for $[M+H]^+$=435.2251, found=435.2241, error=2.30 ppm.

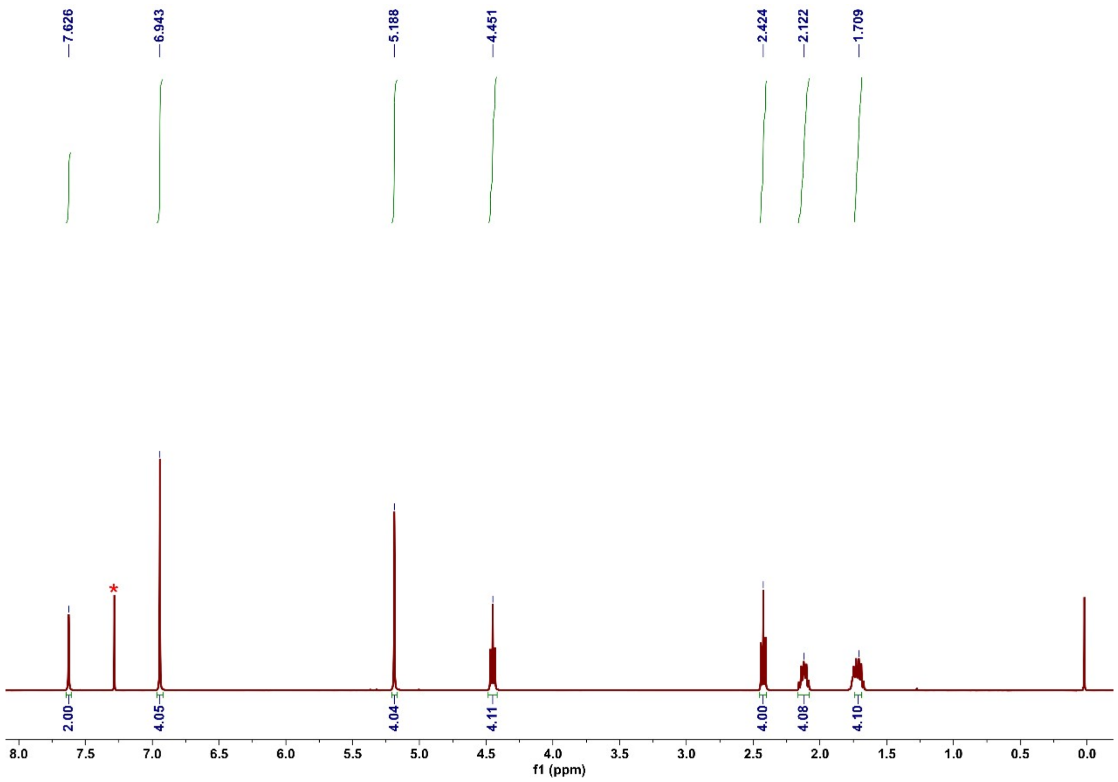

Fig. S49 ^1H NMR spectrum (400 MHz, $CDCl_3$, 298 K) of compound G2

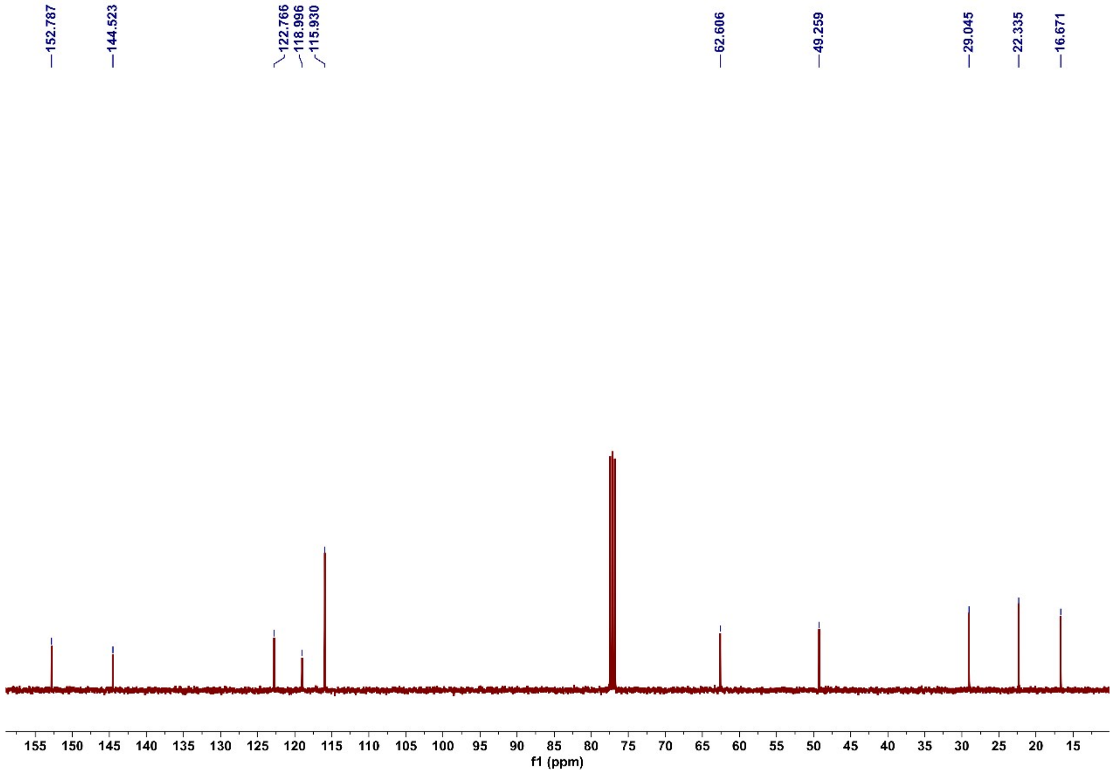

Fig. S50 ^{13}C NMR spectrum (400 MHz, $CDCl_3$, 298 K) of compound G2

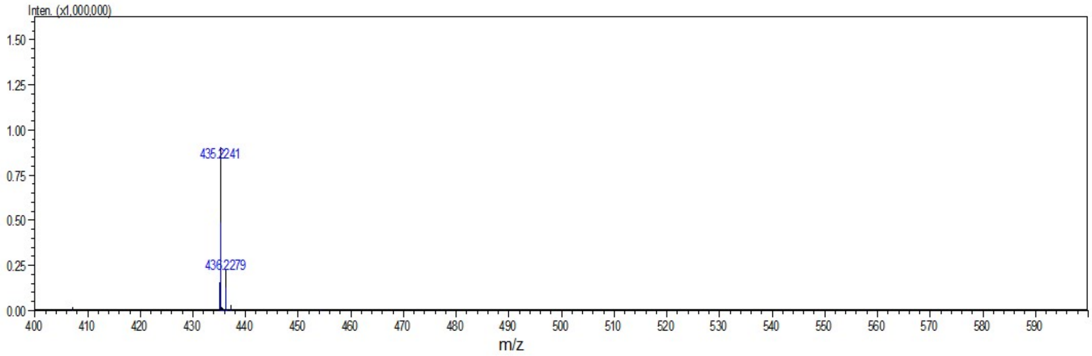

Fig S51 High-resolution HR-ESI-MS mass spectrum of compound G2

References

[S1] H. W. Gibson, N. Yamaguchi and J. W. Jones. Supramolecular Pseudorotaxane Polymers from Complementary Pairs of Homoditopic Molecules. *J. Am. Chem. Soc.* 2003, **125**, 3522–3533.

[S2] W, Likussar and D. F. Boltz. Spectrophotometric determination of extraction constants for certain metal 1-pyrrolidinecarbodithioates. *Anal. Chem.*, 1971, **43**, 1273-1277.

[S3] H. Li, S. H. Rao, Y. Yang, F. F. Xu, Z. Huang, X. H. Huang, Z. Zhu, S. Y. Liu, Z. L. Zhang and W. Tian. Multistep sequence-controlled supramolecular polymerization by the combination of multiple self-assembly motifs. *iScience*, 2023, **26**, 106023.

[S4] S. Y. Wang, J. H. Fu, Y. P. Liang, Y. J. He, Y. S. Chen and Y. T. Chan, Metallo-Supramolecular Self-Assembly of a Multicomponent Ditrigon Based on Complementary Terpyridine Ligand Pairing, *J. Am. Chem. Soc.,* 2016, **138**, 3651−3654.

[S5] Y. J. He, T. H. Tu, M. K. Su, C. W. Yang, K. V. Kong and Y. T. Chan, Facile Construction of Metallo-Supramolecular P3HTb-PEO Diblock Copolymers via Complementary Coordination and Their Self-Assembled Nanostructures, *J. Am. Chem. Soc*., **2017**, 139, 4218–4224.

[S6] T. Ogoshi, T. Aoki, K. Kitajima, S. Fujinami, T. Yamagishi and Y. Nakamoto. Facile, rapid, and high-yield synthesis of pillar [5] arene from commercially available reagents and its X-ray crystal structure. *J. Org. Chem*., 2011, **76**, 328-331.

[S7] Z. Zhang, H. Wang, X. Wang, Y. M. Li, B. Song, O. Bolarinwa, R. A. Reese, T. Zhang, X. Q. Wang, J. F. Cai, B. Q. Xu, M. Wang, C. L. Liu, H. B. Yang and X. P. Li. Supersnowflakes: stepwise self-assembly and dynamic exchange of rhombus star-shaped supramolecules. *J. Am. Chem. Soc*., 2017, **139**, 8174-8185.

[S8] H. Li, W. Z. Chen, F. F. Xu, X. D. Fan, T. X. Liang, X. P. Qi and W. Tian. A Color-Tunable Fluorescent Supramolecular Hyperbranched Polymer Constructed by Pillar [5] arene-Based Host–Guest Recognition and Metal Ion Coordination Interaction. *Macromol. Rapid Commun*., 2018, **39**,1800053.
